# Observation of a supersolid phase in a spin-orbit coupled exciton-polariton Bose-Einstein condensate

**DOI:** 10.1126/sciadv.aee4875

**Published:** 2026-10-16

**Authors:** Marcin Muszyński, Pavel Kokhanchik, Rafał Mirek, Darius Urbonas, Pietro Tassan, Piotr Kapuściński, Przemysław Oliwa, Ioannis Georgakilas, Thilo Stöferle, Rainer F. Mahrt, Michael Forster, Ullrich Scherf, Dmitriy Dovzhenko, Rafał Mazur, Przemysław Morawiak, Wiktor Piecek, Przemysław Kula, Barbara Piętka, Dmitry Solnyshkov, Guillaume Malpuech, Jacek Szczytko

**Affiliations:** ^1^ Institute of Experimental Physics, Faculty of Physics, University of Warsaw, ul. Pasteura 5, 02-093 Warsaw, Poland.; ^2^ Université Clermont Auvergne, Clermont Auvergne INP, CNRS, Institut Pascal, F-63000 Clermont-Ferrand, France.; ^3^ IBM Research Europe—Zurich, Säumerstrasse 4, 8803 Rüschlikon, Switzerland.; ^4^ Macromolecular Chemistry Group and Wuppertal Center for Smart Materials & Systems (CM@S), Bergische Universität Wuppertal, Gauss Strasse 20, 42119 Wuppertal, Germany.; ^5^ School of Physics and Astronomy, University of Southampton, Southampton SO17 1BJ, UK.; ^6^ Institute of Applied Physics, Military University of Technology, ul. gen. Sylwestra Kaliskiego 2, 00-908 Warsaw, Poland.; ^7^ Institute of Chemistry, Military University of Technology, ul. gen. Sylwestra Kaliskiego 2, 00-908 Warsaw, Poland.; ^8^ Institut Universitaire de France (IUF), 75231 Paris, France.

## Abstract

In Bose-Einstein condensates, spin-orbit coupling (SOC) produces supersolidity. It is a peculiar state of matter, which, in addition to the superfluid behavior, shows periodic density modulation typical for crystals. Here, we report the fabrication of an original type of optical microcavity, allowing the achievement of room-temperature supersolidity for a quantum fluid of light. The microcavity is filled with a nematic liquid crystal (LC) and two layers of the organic polymer methyl-substituted ladder-type poly(*p*-phenylene) (MeLPPP) hosting exciton resonances. We demonstrate exciton-polariton condensation in the two distinct degenerate minima of the dispersion created by the LC-induced Rashba-Dresselhaus SOC. The condensate real-space distribution shows density stripes located randomly from one condensate realization to another despite the presence of a random disorder potential. This demonstrates the immunity of stripes against disorder and the spontaneous breaking of translational invariance. We also report the random appearance of vortices, another feature of superfluids.

## INTRODUCTION

Supersolidity is an intriguing state of matter that combines two antagonistic features, namely, solid and superfluid properties. Here, by solid, we mean crystalline order or periodic modulation of a medium. It is combined with a frictionless flow in the presence of rugosity. Supersolidity was first conjectured in helium (*1*) but was found difficult to observe (*2*). The supersolid phase is expected to play a key role in the behavior of neutron stars being a possible explanation for the abrupt changes of their rotation frequency (glitches) (*3*). Bose-Einstein condensates (BECs) of weakly interacting bosons exhibit superfluidity, and different methods have been implemented to break their translational invariance, hoping to preserve at the same time their superfluid properties. The first way was to consider the action of optical lattices, which can even be continuously tuned (*4*). The second way was to engineer a so-called Rashba-Dresselhaus spin-orbit coupling (RDSOC) (*5*, *6*) in a spinor BEC (*7*–*9*). As sketched in Fig. 1A, the RDSOC deeply modifies the mode dispersion, leading to the formation of two degenerate energy minima in the two-dimensional (2D) wave vector space, centered at two opposite nonzero wave vectors *k*_0_. The condensates that form in these two minima interfere, creating density stripes in real space. The wave vector *k*_0_ governs the period, whereas the position of the stripes depends on the two spontaneously broken phases Φ_1_ and Φ_2_. The third way to create a supersolid is to use atoms with dipolar interactions that form periodic droplet patterns, for which superfluidity (*10*, *11*) and even vortices have recently been reported (*12*). Supersolidity has also been suggested in other systems, such as hybrid Bose-Fermi mixtures (*13*). All these experiments were done with ultracold atomic gases at sub-microkelvin temperatures.

**Fig. 1.**
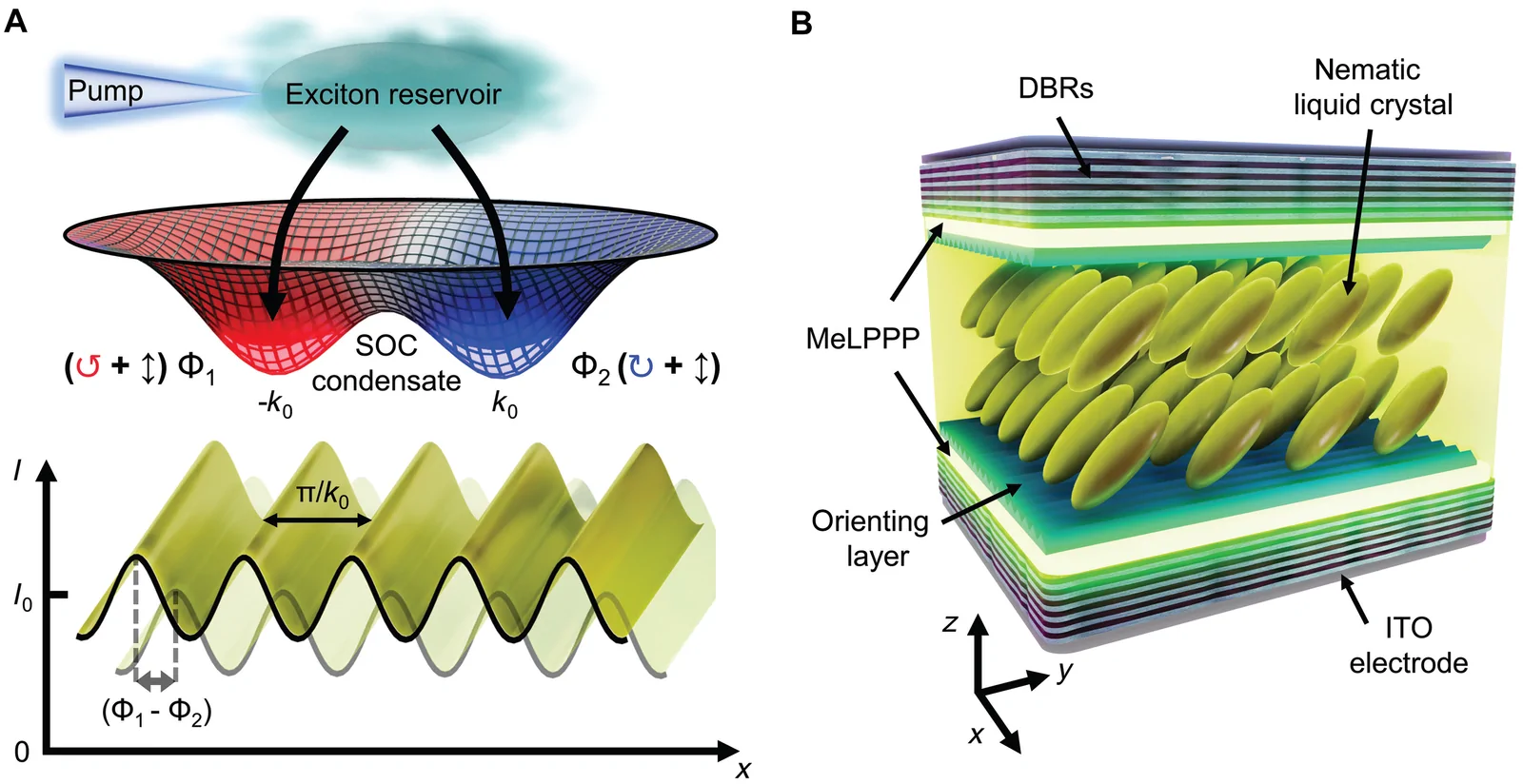
SOC condensate and microcavity scheme. (**A**) SOC condensate forms with different elliptical polarizations and phases Φ_1_ and Φ_2_ in the two dispersion minima. Their interference results in the formation of density stripes in real space with position depending on Φ_1_ − Φ_2_. (**B**) Microcavity scheme: Thick LC layer sandwiched between two organic polymer layers (MeLPPP), two DBRs, and two ITO electrodes.

In addition to cold atoms, photonic platforms, such as microcavities and photonic crystals, also host macroscopically populated states of interacting bosons [exciton-polaritons (*14*, *15*)]. Superfluidity was demonstrated under resonant excitation (*16*, *17*) by making a flow hit a defect without being perturbed. Under nonresonant excitation, algebraic decay of coherence compatible with the Berezinskii-Kosterlitz-Thouless picture of superfluidity was reported (*18*). Density stripes have also been recently reported at both cryogenic (*19*) and room temperature (*20*, *21*) in polaritonic crystal slabs hosting bound states in the continuum with negative effective mass, in which bright soliton condensates are formed (*22*–*24*). The polaritonic modes were organized in such a way as to allow the condensate depletion by resonant parametric scattering. As a result, the soliton condensate interferes with the signal and idler being at the same energy, which leads to a spatially modulated amplitude of the wave function.

Photonic platforms also represent paradigmatic systems to engineer 2D photonic modes demonstrating topological singularities and topological transitions (*25*–*39*). The key ingredient behind this richness is the different types of effective SOC of light, but also the full tomographic access to the particle wave functions (*32*). The most prominent SOC, which was described quasi-simultaneously in microcavities (*25*) and in other systems (*40*), is related to the transverse-electric/transverse-magnetic (TE-TM) nature of light modes. Another one is an emergent RDSOC, which was recently implemented in microcavities (*31*). It occurs when Fabry-Pérot modes of different parities and different polarizations are brought in resonance by a large linear birefringence and when the optical axis of the media filling the cavity differs from the one of the cavity itself. In that perspective, the realization of cavities filled with nematic liquid crystals (LCs) (*31*, *34*, *41*, *42*) represented a breakthrough, because it allows to tune linear birefringence and to control RDSOC on demand. A key aspect of RDSOC with respect to TE-TM SOC is that it scales linearly with the wave vector, and therefore, the resulting dispersion shows two degenerate minima at ±*k*_0_. The cavity exciton-polariton platform is therefore ideal for the study of the behavior of quantum fluids (*14*) in the presence of SOC and in topologically nontrivial bands.

The combination of strong exciton-photon coupling with mode tunability including the RDSOC regime has so far been achieved using lead halide perovskites as an active material (*39*, *41*–*43*). These materials suffer from limited stability against photo-excitation. As an alternative, organic polymer methyl-substituted ladder-type poly(*p*-phenylene) (MeLPPP) (*44*) appears quite suitable, as it has demonstrated exciton-polariton condensation at room temperature (*45*, *46*). Its high saturation density, quantum efficiency, and photostability allow single shot measurements, which can be repeated a large number of times at a given sample location.

In this work, we report the fabrication and optical characterization of original microcavities with embedded nematic LC and MeLPPP (scheme shown in Fig. 1B). We prove the formation of strongly coupled exciton-photon modes with Rabi splittings of the order of 90 meV. We demonstrate the tunability of these modes in a wide spectral range by an external electric field controlling the linear birefringence, thereby reaching the RDSOC regime in which polariton condensates demonstrate density stripes. In single shot experiments and for sufficiently large condensate densities, we observe the stripe patterns placing themselves randomly from one condensation process to another, in agreement with theoretical simulations. This demonstrates the spontaneous breaking of translational invariance. The preservation of spontaneous symmetry breaking despite the presence of the disorder potential of the samples is a typical feature of a superfluid. For smaller condensate densities (close to threshold), we find both theoretically and experimentally the stripes to be partly pinned by disorder, suggesting a non-superfluid behavior. We also observe the presence of quantized vortices in about 10% of the condensate realizations.

## RESULTS

### Microcavity fabrication and characterization

A scheme of the fabricated samples is shown in Fig. 1B (sample preparation details are given in Materials and Methods). A typical sample consists of SiO_2_/Ta_2_O_5_ distributed Bragg reflectors (DBRs), which form a Fabry-Pérot resonator hosting a series of longitudinal modes labeled by their integer order. A nematic LC is embedded within the cavity. When a voltage is applied to the electrodes, the LC molecules rotate in the *yz* plane defined by orienting layers, thus providing a tunable birefringence. The LC refractive indices along the ordinary and extraordinary axes are *n*_o_ ≈ 1.57 and *n*_e_ ≈ 1.98, respectively. Modes polarized along *y* under normal incidence (H) are tunable by LC rotation, while modes polarized along *x* (V) are not affected by voltage. However, both can be tuned by changing the position on the sample thanks to a slightly wedged cavity. Two 35-nm-thick layers of MeLPPP (*M*_n_ = 31,500, *M*_w_ = 79,000) surround the LC layer. Details on the optical response of MeLPPP excitons are provided in Materials and Methods (fig. S1) and in (*45*, *47*). We fabricated a series of similar samples, because the optical quality of the MeLPPP, in spite of its high photostability, was found to degrade in time, probably due to interplay with LC molecules, with a scale ranging from a few days to a few weeks.

The samples were analyzed by angle-resolved photoluminescence (PL) (see Materials and Methods). Figure 2 (A to C) shows, for sample “1,” several of these spectra detected in H polarization. The modes 15H and 14V enter in resonance at 3 V (Fig. 2A), giving rise to the typical “Rashba” spectrum (RDSOC regime). At all voltages, the dispersion and polarization at small *k* are described (black lines in Fig. 2, A to C) by a 2 × 2 Hamiltonian (written on the circular basis) including the RDSOC coupling between H and V (*31*), which reads

$$H_{\text{RDSOC}}=\frac{\hbar^{2}k^{2}}{2M}I+\left(\Delta +\gamma k^{2}\right)\sigma_{x}+2\alpha k_{x}\sigma_{z}$$ (1)

where *k* is a 2D wave vector, *I* is the identity matrix, $\sigma_{x,z}$ are Pauli matrices, $\Delta =E_{V}-E_{H}$ is the H-V detuning (corresponding to Raman coupling in atomic systems), $M=2m_{V}m_{H}/\left(m_{H}+m_{V}\right)$, $\gamma =\hbar^{2}/2m$, $m=2m_{V}m_{H}/\left(m_{H}-m_{V}\right)$, and α is the RDSOC coupling constant.

**Fig. 2.**
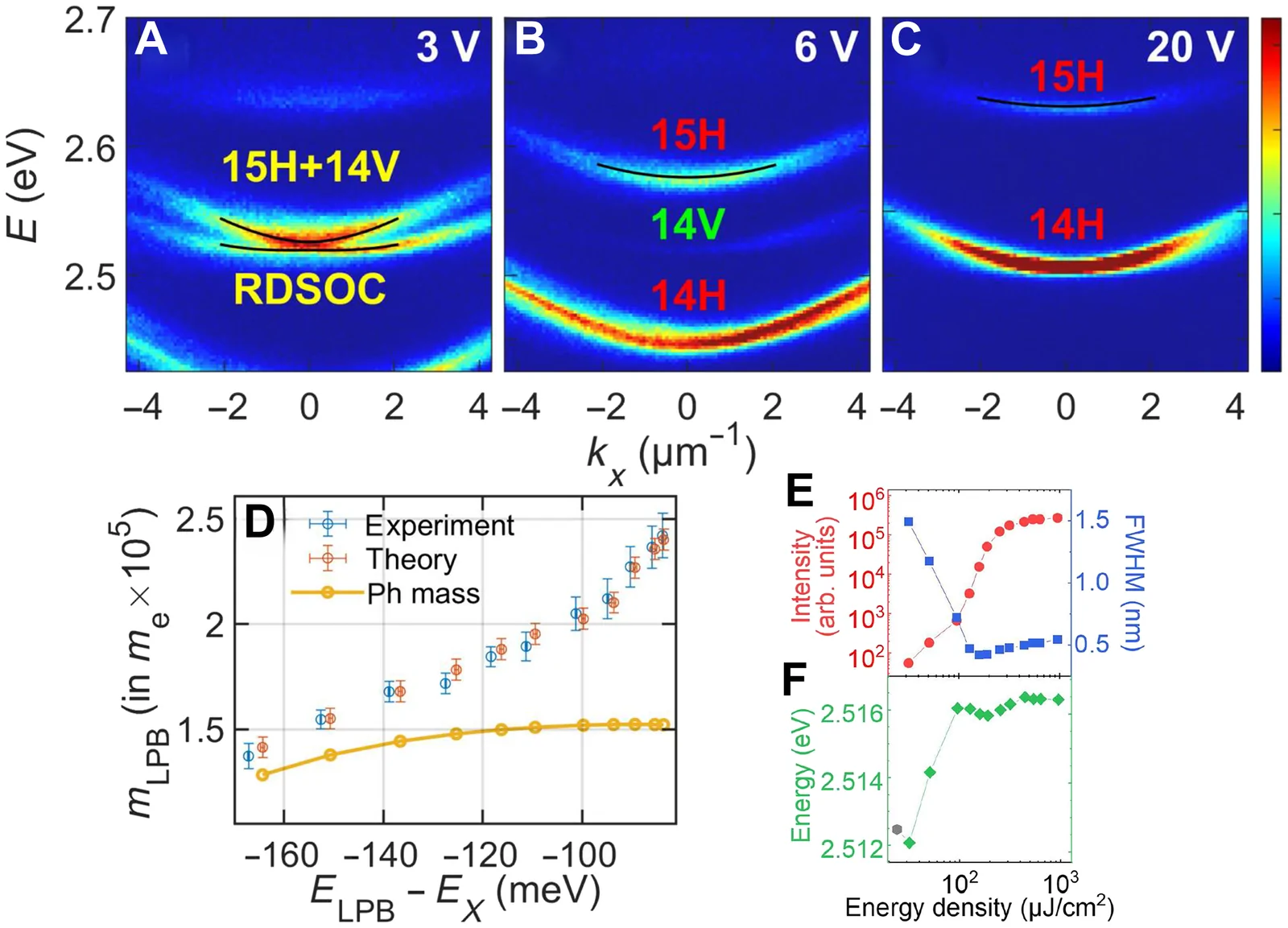
Demonstration of strong coupling regime. (**A** to **C**) Experimentally measured angle-resolved PL for different values of voltage, showing tunability of the H polarized modes and (A) realization of the RDSOC regime; red, green, and yellow text labels the number and polarization of the mode(s); black solid lines show the fitting (A) by Hamiltonian (1) and [(B) and (C)] by parabola; the excitonic resonance is centered at *E_X_* = 2.715 eV. (**D**) LPB mass as a function of LPB detuning: extracted from the experiment (blue dots) and numerical simulation (red dots); the numerical model uses the mode photonic mass at each voltage (yellow dots); the error bars represent the method uncertainty (see the Supplementary Materials). (**E**) Emission intensity (red dots), linewidth (blue squares), and (**F**) mode energy (green diamonds) as a function of the excitation density. An extra point from reflectivity (gray hexagon) added for comparison.

With increasing voltage (Fig. 2, A to C), the mode 15H quits the strong RDSOC regime and approaches the exciton resonance energy *E_X_* = 2.715 eV from below. We see in this figure and all other samples as well that the mass of the modes as they approach the exciton is strongly increasing, indicating strong mixing of the photon with the exciton resonance. The mass of this lower polariton branch (LPB) is extracted by fitting the PL with a parabola, shown by the black solid line. The mass dependence versus LPB detuning *E*_LPB_ − *E_X_* (controlled by voltage) is displayed in Fig. 2D. To fit this experimental dependence and extract the value of Rabi splitting Ω, we use a Tavis-Cummings model (see Materials and Methods), taking into account the inhomogeneous broadening for excitons in MeLPPP. The model includes a single photonic mode, defined by its mass and energy obtained from the Berreman method simulation (see the “Photonic mass” section), and *N* excitons, having a Gaussian distribution of their energies and each coupled to a photonic mode with the coupling strength $\hbar g=\Omega /\sqrt{N}$. Therefore, for each voltage, our theoretical model provides both the LPB energy and mass, which we plot in Fig. 2D. The extracted Rabi splitting is Ω = 93 meV. The constructed model is equivalent to the coupled oscillator model with two oscillators coupled by Ω for large exciton-photon detunings, while the behavior of the two models diverges when the detuning is reduced. We also extracted the LPB exciton fraction of ≈5.5% in the RDSOC regime (Fig. 2A). These results confirm the achievement of exciton-photon strong coupling in the presence of RDSOC at negative exciton-photon detuning. This feature is robust and qualitatively repeated for all samples studied in this work. At low detunings, an anticrossing can be observed in a part of the samples (see the “Anticrossing” section), but not always, because of the cumulative inhomogeneous and temperature-induced broadening comparable with the Rabi splitting Ω. Nevertheless, in all samples, the modes are strongly coupled exciton-photon states at negative exciton-photon detuning (see Materials and Methods).

### Observation of stripe phase

We now consider strong optical pumping for which room temperature exciton-polariton condensation occurs. We use pulsed nonresonant excitation (see the “Experimental details” section), and our observations are based both on multiple-shot experiments averaging over 500 to 2500 pulses and on single-shot ones, for which only measurements above the threshold provided a sufficient signal. First, we consider well-separated linearly polarized modes. Figure 2 (E and F) shows, for one set of parameters, the PL emission intensity, linewidth, and energy of the V-polarized polariton mode versus pumping density. The exponential increase in emission intensity, the narrowing of the linewidth, and the weak blue shift provide evidence of polariton condensation. The threshold density of 95 μJ/cm^2^ is comparable to previous reports of MeLPPP polariton condensates (*45*, *47*, *48*). Polariton condensation is assisted by a vibron resonance and occurs about 200 meV below the excitonic peak in modes with 5% exciton fraction. The blue shift of 4 meV is almost three times lower than the 11-meV difference between the LPB and bare photon energies (exciton-photon detuning of −190 meV). This demonstrates that the strong coupling regime holds above the condensation threshold. Also, spatial coherence measurements in the parabolic regime are shown in fig. S5 (and commented in Materials and Methods). Again, these features are robust from one sample to another.

In the next part, we study the condensation in the RDSOC regime, first in multiple-shot experiments (500 shots). Figure 3A shows the k-space emission just above the condensation threshold at 0 V, when linearly polarized modes are well separated, while the corresponding real-space emission is shown in Fig. 3C. It is affected by disorder, showing bright spots on a homogeneous background of 25 μm diameter, comparable with the pump spot size. The characteristic amplitude of the disorder potential acting on polaritons is given by the inhomogeneous linewidth of the mode emission below the threshold, which is a few milli–electron volts. Fluctuations are of micrometer size. A microscopic image of the sample together with the spatially resolved energy spectrum is shown in fig. S7. The emission is fully linearly polarized (Fig. 3E). The Fourier transform of the real space density (Fig. 3G, dashed line) demonstrates the absence of any spatial periodicity.

**Fig. 3.**
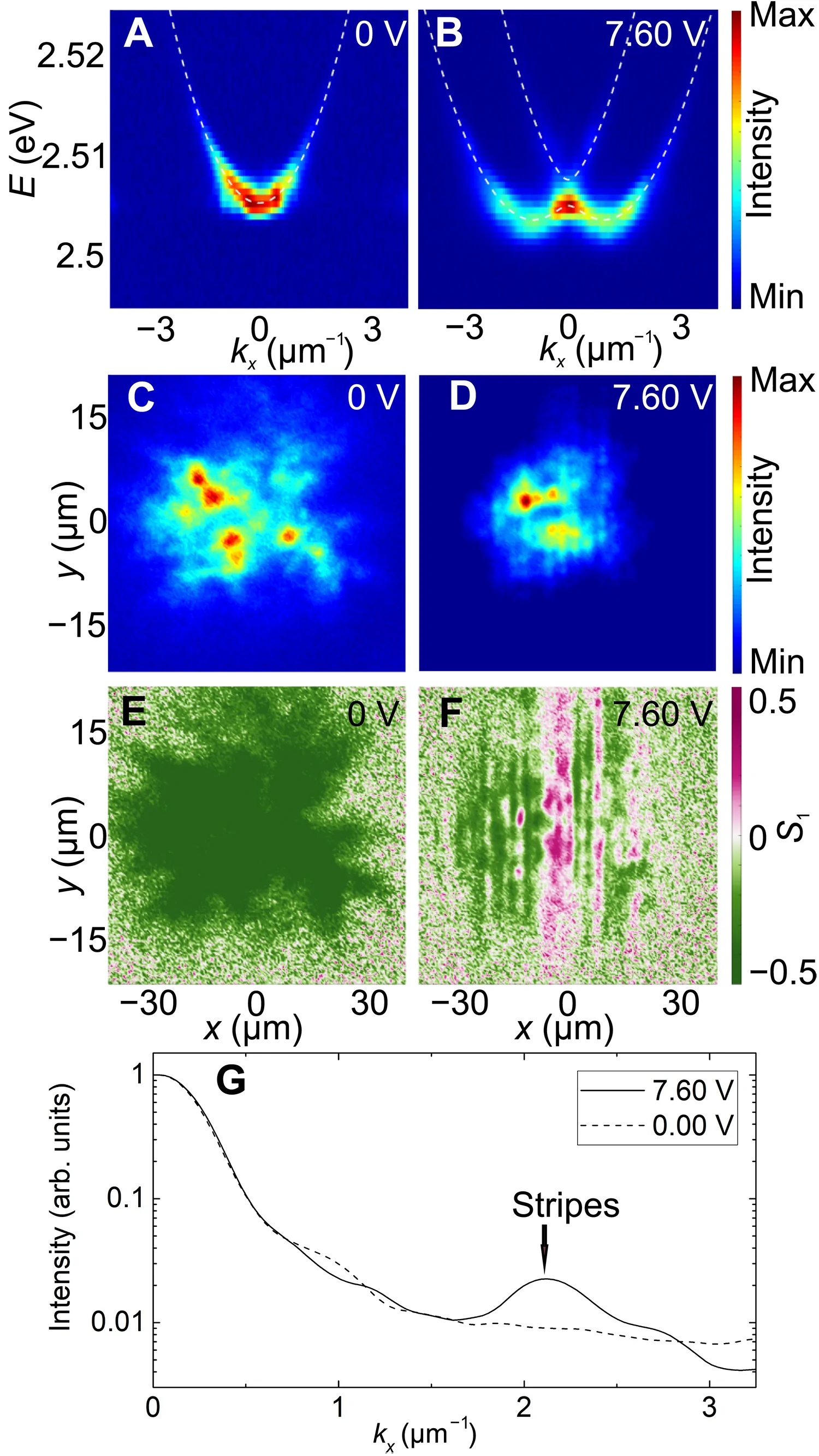
Stripe phase in a polariton condensate. Photoluminescence measured at 0 V (first column) and 7.6 V in the presence of RDSOC (second column). The first row (**A** and **B**) shows the *k_x_*-dependent total emission with the double minima dispersion in (B). The second row (**C** and **D**) shows the real space total emission with stripes absent in (C) and present in (D). The third (**E** and **F**) row shows the real space distribution of the linear *S*_1_ polarization degree. (**G**) Fourier transform of the experimental intensity from (C) and (D) with a marked stripes diffraction peak.

By changing voltage (on the same sample point), two modes of opposite parity come into resonance, turning on the RDSOC (Fig. 3B). The bands show two minima at ±*k*_0_ ≈ 1 μm^−1^. The dispersion is well described by the effective Hamiltonian (1) with Δ = −1.3 meV and α = 2.7 meV μm. This Hamiltonian yields *k*_0_ as

$$k_{0}\approx \frac{2M}{\hbar^{2}}\sqrt{\alpha^{2}-\frac{\hbar^{4}\Delta^{2}}{4m\alpha^{2}}}$$ (2)

The eigenstates at the two minima are elliptically polarized with opposite circular polarization degree and same linear polarization degree (see the “Analytical description of the stripes and their contrast” section). The experimentally measured linear polarization degree is 16%. Its analytical value is

$$S_{1}=\frac{\Delta +\gamma k_{0}^{2}}{\sqrt{4\alpha^{2}k_{0}^{2}+{\left(\Delta +\gamma k_{0}^{2}\right)}^{2}}}$$ (3)

yielding 23%. If one considers an equilibrium BEC forming in these two degenerate dispersion minima, the interference between these components generates polarization stripes (spin helix), whose contrast is given by the circular polarization degree (≈80%), and density stripes with a contrast given by the linear polarization degree (≈16%). The stripes period is theoretically given by π/*k*_0_. If the stripes are located completely randomly from one experiment to another, the contrast of an averaging over *N* realizations should be reduced by $1/\sqrt{N}$ (see section S3), which would give 0.7%.

The actual real space emission (Fig. 3D), besides being affected by disorder (as at 0 V), indeed shows vertical stripes parallel to the *y* axis. The regularity of this modulation is demonstrated in Fig. 3G where the Fourier transform of the real space emission (solid line) demonstrates a peak at *k_x_* = 2*k*_0_ ≈ 2.1 μm^−1^, in reasonable agreement with the one expected from direct k-space measurements. Please note that the symmetric part of the Fourier transform for *k* < 0 is not shown. Also, as explained in more details in the next section, the spatial extension of stripes sets a minimal value for the coherence length of the condensate wave function. Section S1 shows stripes measured at another point with a slightly different voltage. The ratio between the intensity of the diffraction peak and the one at *k_x_* = 0 gives an estimate of the stripes contrast (2.2%), which is three times more than the expected theoretical value, demonstrating the occurrence of a moderate pinning mechanism, either by the pump itself or by the disorder potential. The polarization stripes period (Fig. 3F) is similar to the one of the density stripes, whereas their contrast is 40%.

### Spontaneous breaking of translational invariance, spatial coherence, and superfluidity

We next present in Fig. 4 the results of measurements on sample “2” and sample “3,” similar to sample “1” and all being in the strong coupling regime. The experiments performed were single-shot, with a pulse duration of about 50 ps, much longer than all characteristic timescales, and much longer than for the measurements displayed in Fig. 3. Figure 4 (A and B) shows a typical k-space emission measured on sample 2 in the RDSOC and parabolic regimes, respectively (log color map). One can observe the presence of either one maximum or two maxima of emission in k-space. The k-space distance between the two peaks of the condensate emission Δ*k* = 2*k*_0_ versus voltage is shown in Fig. 4C (black dots) and is in full agreement with the theoretical expectations (blue line, see section S2). The full width at half maximum of each of the two peaks is Δ*k* ≈ 0.5 μm^−1^, which sets a minimal coherence length of 2π/Δ*k* ≈ 12 μm for each of the two condensate components. The energy-dependent emission of the noncondensed part corresponding to the data shown in Fig. 4A is thermal, with a temperature of 313 ± 45 K (see fig. S13). Figure 4 (D and E) shows the real space emission for two different shots, in the RDSOC regime, above threshold. We plot the emission of one linear polarization, for which the contrast is expected to be 100% (see the “Analytical description of the stripes and their contrast” section), and stripes are now extremely clear. Stripes measured from the full density on sample “3,” with a lower contrast, are shown in section S3. Stripes are the result of the interference between the two k-components of the condensate. A comparison of the momentum-space and real-space images shows that each is the Fourier transform of the other. Spatial fluctuations of the phases (ϕ_1_, ϕ_2_) of the two components (see Eq. 5) would destroy this interference and would result in the presence of a large number of dislocations. The observation of stripes therefore directly proves that ϕ_1_ − ϕ_2_ is constant over the condensate’s spatial and temporal extension. Now, comparing Fig. 4 (D and E), one can see that the two realizations are different, with the most intense stripes being approximately in antiphase with each other (the spatially averaged values of the phase are −0.47π and +0.48π, respectively). We access the phase (ϕ_1_ − ϕ_2_), characterizing the position of the stripes, and their contrast by making a Fourier transform of the spatial images. Figure 4F shows histograms of phase distributions over 500 realizations corresponding to a high-density condensate regime for the two left panels, and to a condensate density approximately four times smaller for the right panel. The phase in the high-density regime exhibits uniform distribution in both sample “2” and sample “3,” which suggests the absence of pinning and fully demonstrates the spontaneous breaking of translational invariance. A similar behavior has just been reported in an atomic condensate with SOC (*9*) and in a photonic BIC condensate with parametric coupling to signal and idler (*20*). In contrast, the low-density regime shows a preferential phase corresponding to a partial pinning of stripes in different realizations. One should note that all the data are taken close to threshold. They correspond to a change in the pump density by only 10 to 20%. Another view of the same effect is plotting the contrast of the integrated image versus the number of integrated shots *N* (up to 500; see Fig. 4G). In the high-density regime, the contrasts measured on both sample “2” and “3” decay approximately as $1/\sqrt{N}$, as expected for completely random phases (see section S4). This spontaneous translational symmetry breaking appears to be insensitive to the random disorder present in the samples.

**Fig. 4.**
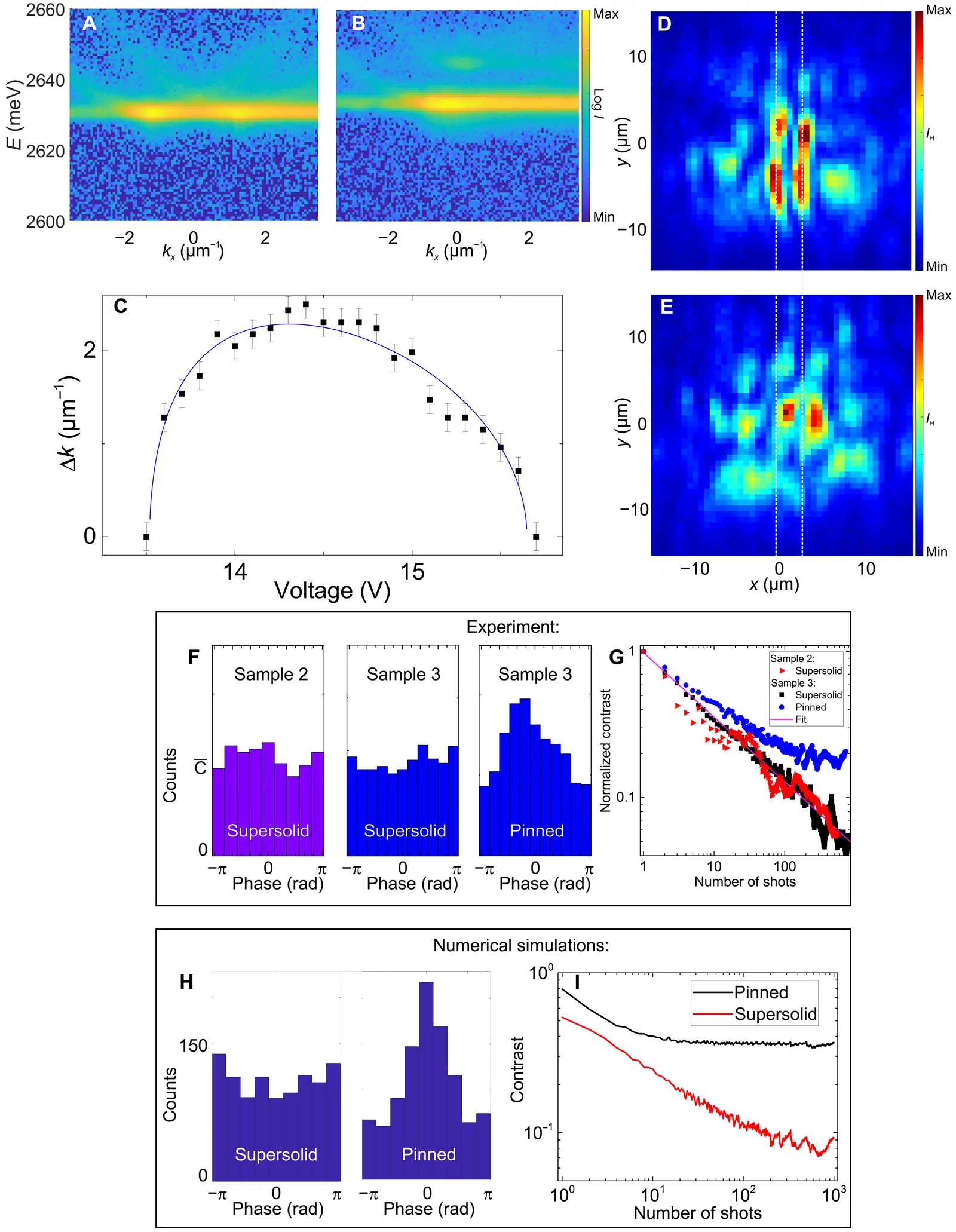
Spontaneous symmetry breaking and superfluidity evidence in single-shot experiments and simulations. (**A** and **B**) k-space saturated emission at 14.5 and 13.5 V, respectively. (**C**) k-space spacing between the emission maxima versus voltage. Error bars represent the experimental uncertainty. The blue line is the theory (see section S2). (**D** and **E**) Two single-shot images of the condensate emission with a linear polarizer in front of the camera. White dashed lines are guides for the eyes, allowing one to visualize the shift of the fringes due to the spontaneous symmetry breaking (random phase). (**F**) The histogram of the values of the phase of the fringes pattern. The two left histograms are taken for high condensate density and show a near-uniform distribution. The right panel is taken at lower density and shows a peak. (**G**) The decrease of the contrast due to averaging for integrated multiple-shot experiments. Red corresponds to the left panel. Black corresponds to the central panel, and blue corresponds to the right panel. The contrast decays as $1/\sqrt{N}$ at large *N*. (**H** and **I**) Simulations. (H) Phase histogram for interacting (left) and noninteracting condensate (right). Interaction energy is 1 meV and disorder amplitude is 0.5 meV. (I) Contrast averaged over multiple shots versus number of shots, with interactions (red) and without them (black).

In contrast, for lower condensate densities, the contrast does not decrease further after averaging of about 10 realizations, which means that stripes choose a preferential location. As one can qualitatively expect, a superfluid behavior of the stripes is favored by a large density and large interaction energy comparable with the disorder amplitude and with the kinetic energy involved in the stripe phase, but also in the dynamical process of condensate formation. This partial pinning just above threshold also explains why we can still observe stripes in multiple-shot experiments with averaging over 500 shots.

A characteristic of superfluids is to flow in a disordered medium without dissipation (*49*, *50*), remaining in an apparently nonequilibrium state, as opposed to a normal fluid, which ultimately localizes. The experiment we realize here is different in the sense that the suppression of the sensitivity of the phase of the condensate to disorder with increasing fluid density occurs not for a flow, but in the course of the condensation process.

We simulate polariton condensation under nonresonant pumping using the hybrid Boltzmann-Gross-Pitaevskii equation with lifetime, energy relaxation, saturated gain (*51*), spin-anisotropic polariton-polariton interactions, and disorder. All details on the model and parameters used are given in Materials and Methods. Figure 4 (H and I) shows the phase histogram and contrast decay versus *N* for two different condensate densities. They reproduce both the pinning effect of the disorder at low densities and the random positioning of the stripes when the interaction energy in the condensate becomes comparable with the disorder magnitude. A discussion about the expected magnitude of interactions in this type of structure is given in section S5. The main mechanism is the screening of the exciton oscillator strength. In section S6, we present simulations performed for continuous-wave pumping, where we compute the dispersion of elementary excitations (bogolons) of the stripe phase, as previously done for equilibrium atomic condensates (*52*) (we do not measure this dispersion experimentally, because the signal from the condensate is too strong). The difference is that here, we model a system with pump, decay, and possibly disorder. These simulations show the existence of gapless linearly dispersing bogolons. However, the presence of two valleys imposes an extra constraint for the existence of stationary superfluid, which is the absence of disorder-induced intervalley scattering. Superfluidity is therefore entirely preserved only if the moving defect is smooth enough, providing elastic scattering on a range smaller than *k*_0_ (*53*, *54*). We point out that such behavior cannot be achieved for stripe condensates composed of negative mass particles (*19*), which form bright solitons.

### Observation of quantum vortices in a supersolid

Another effect that affects the stripe patterns is the possibility of formation of different regions with different relative phases, leading to the formation of vortices. Random creation of vortices typically takes place during the condensation process via the Kibble-Zurek mechanism (*55*, *56*). In an ultracold atomic supersolid, they were recently generated by putting the condensate in rotation (*12*). In Fig. 5 (A and B), we show an example of such a topological defect, which spontaneously appears in the condensation process as a dislocation in the pattern of fringes observed for a particular single-shot image (Fig. 5A), and the associated phase extracted from the Fourier transform (Fig. 5B), exhibiting a characteristic vortex close to the origin of the coordinates (the uncertainty on the vortex position is of the order of the period of the stripes). The results of simulations showing a similar effect are shown in Fig. 5 (C and D). Section S7 presents movies showing the time evolution of stripes during the condensation process. This allows one to observe quantum vortex (dislocation) creation and motion, as well as the dynamic behavior of stripes, even in the presence of disorder. In experiments, vortices are observed at random positions, so they are not directly related to or pinned by the disorder potential. They are observed in about 10% of experiments, with the probability depending on the pumping power. They also affect the decay of contrast with *N* (see section S4). The exact scaling of the vortex number (density) versus the pumping power, which is the quench parameter, and the compatibility of this scaling with the Kibble-Zurek mechanism are presented elsewhere (*57*).

**Fig. 5.**
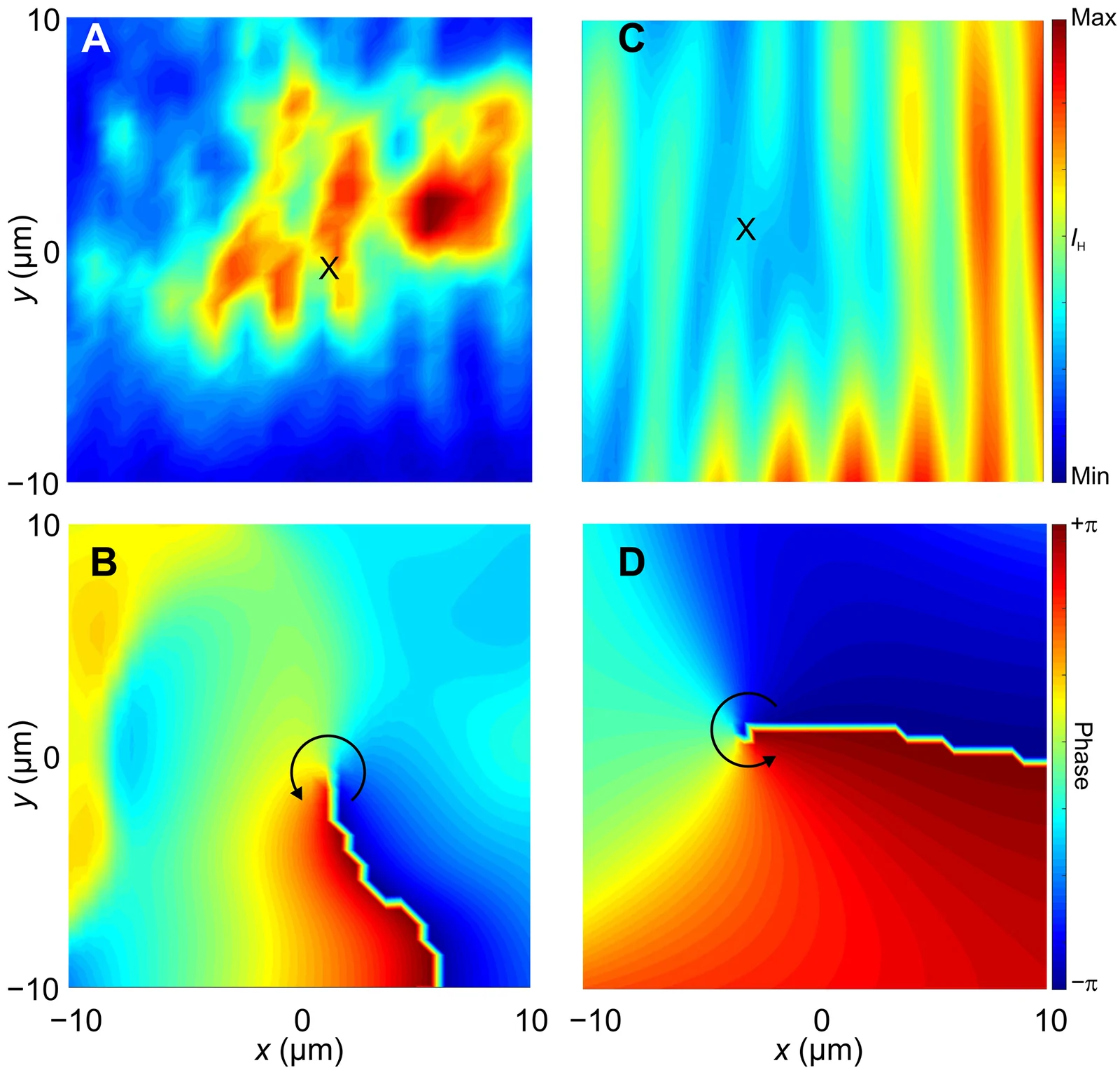
Dislocations and relative phase vortices in single-shot experiments and simulations. (**A** and **C**) A single-shot image with the stripes showing a dislocation (marked by a cross). (**B** and **D**) The extracted phase of the fringes exhibits a vortex singularity. Arrow marks the phase gradient. [(A) and (B)] Experiments; [(C) and (D)] simulations.

## DISCUSSION

In summary, we demonstrated strong light-matter coupling in an optical microcavity with embedded LC and photostable MeLPPP polymer. We observed polariton condensation first in the regime where photonic bands of the cavity are well separated and we demonstrated the tunability of the condensate polarization by either the external voltage or the pump polarization. In the RDSOC regime, the condensate forms in the two degenerate minima of the dispersion. The real space distributions of polarization and intensity reveal the presence of stripes. By single-shot experiments, we observe that polarization stripes are located randomly from one condensate realization to another, demonstrating the spontaneous character of the translational invariance symmetry breaking. The robustness of this process against the intrinsic disorder potential of the structure together with the detection of quantum vortices suggests the superfluid behavior of the stripe phase, even if this last aspect would require further quantitative analysis. These observations confirm the supersolid nature of the observed phase. Our work opens new perspectives for quantum fluids of light physics in the presence of SOC and beyond, in topologically nontrivial systems, both Hermitian and non-Hermitian, at room temperature.

## MATERIALS AND METHODS

### Sample preparation

For all samples, the cavity consists of two DBRs composed of 15 and 13 SiO_2_/Ta_2_O_5_ layers, with a central wavelength at 490 nm. The DBRs were deposited on 30-nm ITO electrode layers on quartz substrates with a flatness of λ/20 (at 633 nm). Both DBRs were spin coated with 35-nm MeLPPP layers covered with protective Al_2_O_3_ layers of 20 nm thickness. Antiparallel orienting layers (SE-130, Nissan Chem., Japan) were deposited on both substrates using the spin-coating method. The wedge cavity was achieved using glass spacers with sizes of 1.5 to 2 μm placed between the substrates. The cavity was filled with an LC mixture, LC2091* (refractive indices *n*_o_ ≈ 1.57 and *n*_e_ ≈ 1.98, for the sodium line 589 nm), in the nematic phase by capillary action.

### Experimental details

Figure S1 presents the optical experimental setup used in multiple-shot experiments. The optical measurements were performed at room temperature. Reflectivity measurements used a microscope objective with 50× magnification, numerical aperture (NA) = 0.65 for both the excitation and the collection of light. The spot had a diameter of approximately 10 μm on the sample. In PL measurements, an additional lens *f* = 100 mm was used to focus the excitation laser onto the 20-μm spot on the surface of the sample. The 181-fs pulsed laser of 425 nm wavelength and repetition rate of 500 Hz was used for nonresonant excitation. Depending on the selected lenses (k-space or r-space) and the light source, the setup allowed measurements in both reciprocal and real space at the same position on the sample. Two sets of a quarter–wave plate, a half–wave plate, and a linear polarizer were used to control the polarization of the excitation and collected signal. To tune the dispersion relation of the cavity modes, the LC microcavity was addressed by an AC waveform generator with a 1010-Hz square signal and varying amplitudes of 0 to 20 V. Paths of white light are indicated by yellow lines, the laser light path is marked with a blue line, and the signal from the sample is represented by a cyan line.

Single-shot images were collected using a modified experimental setup compared to fig. S1. The LC microcavity was addressed by an AC waveform generator with a 1000-Hz square signal that is phase synchronized with the excitation laser pulses. The condensate was nonresonantly excited with pulses at 400 nm wavelength. Coupling the 150-fs pulses into a 25-μm multimode fiber allows us to temporarily stretch them to several tens of picoseconds (about 50 ps) and homogenize the beam profile and the polarization. The excitation light was focused onto the sample with a 20× objective (NA = 0.42). The condensate emission was collected with a 10× objective (NA = 0.30) and detected in a real-space configuration by a camera with a linear polarizer placed in front.

### MeLPPP absorption and complex refractive index

Large inhomogeneous broadening of the excitonic peak plays a substantial role in the theoretical treatment of the results. To estimate this broadening, we measured the absorption spectrum of a MeLPPP layer on the DBR and made a fit of the main excitonic peak by Gaussian function $\text{exp}\left[-{\left(E-E_{X}\right)}^{2}/2\sigma^{2}\right]$, with *E_X_* = 2.715 eV center of the peak and σ = 35 meV standard deviation of exciton energy distribution (fig. S2A). Because we will be considering only strongly negatively detuned photonic modes, we do not need to take into account higher energy absorption peaks.

Also, we show here the MeLPPP complex refractive index real and imaginary parts (fig. S2B), which will be used in the next section for the Berreman method simulation of microcavity structure. The background refractive index of MeLPPP extracted from *Re*(*n*) dependence is $n_{\text{BG}}=1.7$.

### Photonic mass

Because the mode 15H is tuned over a large range of energies in Fig. 2 (LPB detuning between −188 and −84 meV), it is crucial to estimate how the photonic mass of the mode changes in the tuning process. To do this, we first reproduce the band structure of the microcavity with the use of the Berreman method (*58*). We simulate the structure sketched in Fig. 1B and tune the length of the LC layer and the angle of LC molecules to obtain perfect correspondence between measurement and simulation shown in fig. S3. In the experiment, the PL was measured in H polarization, which is why the H mode is well visible for any voltage applied to the microcavity, while the V mode appears only when it couples to the H mode strongly enough (fig. S3A). The numerical simulation (fig. S3, B and D) is represented by the first Stokes parameter of the system (linear polarization degree) to easily distinguish between H and V modes, appearing as blue and red lines, respectively. After achieving correspondence between the experiment and numerical simulation, we calculate once again the dispersion for the same structure, except that now the MeLPPP refractive index is constant and equal to background one ($n=n_{\text{BG}}$), thus eliminating the exciton from the calculation and making the system purely photonic. Then, we extract the mass and the position of the photonic mode and plot them in fig. S3E. The range of photonic mode detunings $E_{\text{ph}}-E_{\text{ex}}$ corresponds to the LPB detunings from Fig. 2D. One can see that the total change of the photonic mass is approximately 19%. This simulation proves that the much bigger mode effective mass change observed in Fig. 2 can be explained solely by exciton-photon coupling.

### The Tavis-Cummings model

The Tavis-Cummings model describes the system consisting of a single cavity mode coupled to *N* two-level systems, excitons in our case. Usually, the model uses identical excitons, while in our case, the inhomogeneous broadening of the excitonic peak plays an important role in quantitative correspondence with experiment. The model reads

$$\begin{array}{cc} H & =\hbar \left(\omega_{\text{ph}}-i\gamma_{\text{ph}}\right)a^{†}a+\sum_{j=1}^{N}\hbar \left(\omega_{\text{ex},j}-i\gamma_{\text{ex}}\right)\sigma_{j}^{+}\sigma_{j}^{-}\\ & +\hbar g\left(a\sigma_{j}^{+}+a^{†}\sigma_{j}^{-}\right) \end{array}$$ (4)

where $\hbar \omega_{\text{ph}}=\hbar \omega_{\text{ph},0}+\hbar^{2}k_{x}^{2}/2m_{\text{ph}}$ is the energy of a cavity mode $\hbar \omega_{\text{ph},0}$ is the energy at *k_x_* = 0 and $m_{\text{ph}}$ is cavity photon mass; $\gamma_{\text{ph}}$ is cavity mode decay; $\hbar \omega_{\text{ex},j}$ is the energy of the *j*th exciton taken from inhomogeneously broadened Gaussian distribution; $\gamma_{\text{ex}}$ is the nonradiative decay of a single exciton; ℏg is exciton-photon coupling strength; $a^{†}$ and *a* are cavity mode creation and annihilation operators, respectively; and $\sigma_{j}^{+}=e_{j}g_{j}$ and $\sigma_{j}^{-}=g_{j}e_{j}$ are *j*th two-level system raising and lowering operators, respectively. The total Rabi splitting can be estimated as $\Omega =\hbar g\sqrt{N}$ for large exciton-photon detunings.

For every voltage, we extracted the photonic mode energy $\hbar \omega_{\text{ph}}$. Because the characteristic pump spot size (*w*_pump_ ≈ 20 μm) is much larger than the estimated transverse cavity photon coherence length (*l*_coh_ ≈ 1.5 μm), we consider $N_{\text{ens}}=w_{\text{pump}}^{2}/l_{\text{coh}}^{2}$ different realizations of Hamiltonian (4): Each realization differs by excitonic disorder (ensemble of $\hbar \omega_{\text{ex},j}$) and coupled photon. Using extracted photonic data, we diagonalize each realization of the Hamiltonian (4), and for each realization, we extract the eigenmode with the highest photonic contribution. Next, we sum up the PL signal originating from these eigenmodes for all wave vectors. After applying this procedure, we obtain a total PL signal similar to the experimental one. We then extract the mass and energy of the numerically obtained LPB, exactly like we do with the experimentally measured PL intensity maps, and plot the final results in Fig. 2D. As one can see, both LPB detuning and mass obtained by numerical simulation correspond well to the experimental data when we choose Rabi splitting of Ω = 93 meV. The number of excitons used for every realization of Hamiltonian (4) is *N_X_* = 200. In reality, the number of excitons for each coherent spot $l_{\text{coh}}^{2}$ should be much bigger, but increasing this number very quickly makes the Hamiltonian diagonalization computationally heavy. That is why we made sure that the simulation results slowly converge when we increase *N_X_* up to 2000, then we performed all the simulations for *N_X_* = 200 and included the difference as a method error shown by red error bars in Fig. 2D. The decay values used in simulations are *ħ*γ_ph_ = 3 meV and *ħ*γ_ex_ = 0.1 meV (*45*).

### Anticrossing

Figure S4 shows the white light transmission spectrum versus voltage measured on sample “3,” similar to the two samples studied in the main text. However, the H-mode here displays a clear anticrossing with the exciton resonance. It was shown by Kavokin and Kaliteevski (*59*) in 1995 from Maxwell equations that the optical response of a microcavity can be well described by a two–damped oscillator model. Such a model was used by Savona *et al.* (*60*) to assess the weak-to-strong coupling transition. Within this model, the energy splitting between modes reads $\sqrt{{\left[E_{\text{ph}}-E_{x}+i\left(\hbar \gamma_{\text{ph}}-\hbar \gamma_{\text{ex}}\right)\right]}^{2}+{\left(\hbar g\right)}^{2}}$. The fit uses *ħg* = 90 meV, in agreement with the values found using the more accurate Tavis-Cummings model and *ħ*γ_ex_ − *ħ*γ_ph_ = 70 meV. At zero exciton-photon detuning, the same expression reads $\hbar \sqrt{g^{2}-{\left(\gamma_{\text{ph}}-\gamma_{\text{ex}}\right)}^{2}}$. If the broadening is larger than 90 meV, then it leads to the weak coupling regime at zero detuning, where the real parts of mode energies are degenerate. This zero detuning condition is what is nowadays called an exceptional point. When the detuning is nonzero, the square root above acquires a nonzero real part and the eigenmode energy splitting initially scales as a square root of the detuning. It means that everywhere, but at zero detuning, eigenstates are mixed exciton-photon states. However, Savona proposed that the strong coupling should require that the real part splitting exceeds the sum of the imaginary parts corresponding to the mode linewidths. It means that two resonant peaks should be distinguishable in the optical spectroscopy response of the cavity.

This condition is realized by far when the exciton-photon detuning $|E_{\text{ex}}-E_{\text{ph}}|$ exceeds the difference between the mode linewidths $|\hbar \left(\gamma_{\text{ph}}-\gamma_{\text{ex}}\right)|$. The system can therefore be weakly coupled at zero detuning, and strongly coupled at nonzero detuning, which leads to the very strong mass change experimentally observed and well described by the Tavis-Cummings model, which is the most appropriate to describe systems with large inhomogeneous broadening.

The Rabi splitting value controls the level of mixing that can be realized at the detuning where strong coupling takes place. At a detuning of −200 meV, as the one for which the condensation is taking place, the exciton fraction is between 5 and 10% and only marginally depends on whether the system is weakly or strongly coupled at zero detuning, (i.e., if the broadening is 70 or 95 meV, for instance).

### Rashba-Dresselhaus spin-orbit coupling

The emergence of the RDSOC in microcavities with strong birefringence has been found experimentally and described theoretically using an effective Hamiltonian derived from the Maxwell’s equations in (*31*) (the optical axis of the birefringent material needs to be tilted away from the growth axis). It stems from the chirality of the system as a whole: If one represents the two mirrors and the optical axis by three lines, the structure that they form, from the point of view of a beam with a nonzero in-plane wave vector, looks like a letter Z, different from its mirror image—such structures have been used in photonics to create chiral metamaterials (*61*). A more strict mathematical interpretation is based on the fact that the active region with a tilted optical axis acts like a λ/2 plate, flipping the linear polarization about a given axis. A mirror also flips the linear polarization about a different axis. The net result is the rotation of the linear polarization by a double of the angle between the two mirror planes (*33*). Because the linear polarization is always rotated, it cannot be an eigenstate, and therefore, the eigenstate is circular polarized. A deeper symmetry analysis of the problem has been carried out in (*62*).

### Interference measurements

Figure S5 shows the experimental interferogram of a single realization of the polariton condensate measured at an excitation intensity of around 1.2 *P*_th_ with a Michelson interferometer. To qualitatively demonstrate the established long-range spatial coherence, we overlapped on the camera the original and inverted real-space image of the condensate emission going through the separate arms of the Michelson interferometer at zero time delay in a single-shot regime. A retroreflector was mounted on one of the arms of the Michelson interferometer. The experiment was using a slightly different excitation scheme with respect to that of the main text. We nonresonantly excited the condensate using a 6-ns pulsed laser at a 425-nm wavelength and measured the interferogram in a single-shot regime using the fast external trigger regime on the EMCCD. Interferometry data were acquired at 0 V, so in the regime of parabolic dispersion (RDSOC off), and with a pump spot of 20 μm. In fig. S5, the interference fringes are observed over the whole area of the condensate in the real space, demonstrating a buildup of the long-range spatial coherence, which is a hallmark of the exciton-polariton condensation.

### Analytical description of the stripes and their contrast

The wave function describing a spinor wave function with two contributions corresponding to the eigenstates at two opposite wave vectors ±*k*_0_ can be written in circular basis as

$$|\psi \rangle =\frac{1}{2}\left(\begin{array}{c} \text{cos}\frac{\theta }{2}\\ \text{sin}\frac{\theta }{2} \end{array}\right)e^{-{\mathrm{ik}}_{0}x-i\phi_{1}}+\frac{1}{2}\left(\begin{array}{c} \text{cos}\frac{\pi -\theta }{2}\\ \text{sin}\frac{\pi -\theta }{2} \end{array}\right)e^{+{\mathrm{ik}}_{0}x-i\phi_{2}}$$ (5)

where θ is the polar angle of the Stokes vector, whereas the azimuthal angle is fixed by the polarization of the coupled modes (H and V). The phases ϕ_1_ and ϕ_2_ of the two orthogonal components are chosen randomly due to the spontaneous symmetry breaking. Rewriting the expression allows one to make appear explicitly a random phase responsible for the position of the stripes, and another one (an overall global phase) that can be detected in coherence measurements via interference (as in fig. S5, for example)

$$|\psi \rangle =\frac{1}{2}e^{\mathrm{i\zeta}}\left[\left(\begin{array}{c} \text{cos}\frac{\theta }{2}\\ \text{sin}\frac{\theta }{2} \end{array}\right)e^{-i\left(k_{0}x-\eta \right)}+\left(\begin{array}{c} \text{cos}\frac{\pi -\theta }{2}\\ \text{sin}\frac{\pi -\theta }{2} \end{array}\right)e^{+i\left(k_{0}x-\eta \right)}\right]$$ (6)

where the global phase is $\zeta =-\left(\phi_{1}+\phi_{2}\right)/2$, and the relative phase controlling the position of the stripes is $\eta =-\left(\phi_{1}-\phi_{2}\right)/2$.

The corresponding density reads

$$\langle \psi |\psi \rangle =\frac{1}{2}+\frac{1}{2}\text{cos}2\left(k_{0}x-\eta \right)\text{sin}\theta$$ (7)

It exhibits oscillations, whose contrast is *U* = sin θ and whose position is controlled by the randomly chosen phase η. This expression gives a direct relation between the circular (or linear) polarization degree with the contrast of the stripes observed in total intensity. It also allows one to understand the decrease of the contrast in multiple-shot integrated experiments, where the position of stripes in each shot is random (see the Supplementary Materials).

Optical experiments allow one to measure the intensity of a single linear polarization, with its amplitude obtained from the circular components as $\psi_{H}=\left(\psi_{1}+\psi_{2}\right)/\sqrt{2}$. This allows one to strongly improve the contrast, as required for single-shot measurements

$$\begin{array}{cc} I_{H} & =\langle \psi_{H}|\psi_{H}\rangle ={\text{sin}}^{2}\left(\frac{\pi }{4}+\frac{\theta }{2}\right){\text{cos}}^{2}\left(k_{0}x-\eta \right)\\ & ={\text{sin}}^{2}\left(\frac{\pi }{4}+\frac{\theta }{2}\right)\left[\frac{1}{2}+\frac{1}{2}\text{cos}2\left(k_{0}x-\eta \right)\right] \end{array}$$ (8)

The contrast of this signal is always 100% (at least without the experimental background noise), no matter the value of θ, contrary to the stripes observed directly in ${\left|\psi \right|}^{2}$, while the origin and the position of the stripes are exactly the same, governed by the phase η, as can be seen from the cosine function.

### Numerical simulations

To describe the polariton condensation in our system, we solve the following equation (*51*, *63*, *64*)

$$\begin{array}{c} i\hbar \frac{\partial \psi_{\pm }}{\partial t}=\left(1-i\Lambda \right){\tilde{F}}^{-1}\left[\left(\frac{\hbar^{2}k^{2}}{2M}\pm 2\alpha k_{x}-E_{\text{min}}\right)\tilde{F}\left(\psi_{\pm }\right)+\left(\Delta +\gamma k^{2}\right)\tilde{F}\left(\psi_{\mp }\right)\right]\\ +g_{1}{|\psi_{\pm }|}^{2}\psi_{\pm }+g_{2}{|\psi_{\mp }|}^{2}\psi_{\pm }+i\Gamma \left({|\psi |}^{2}\right)\psi_{\pm }+U\psi_{\pm }+\chi \end{array}$$ (9)

Here, $\tilde{F}$ is the Fourier transform (accelerated by the graphics processing unit). It contains terms accounting for several effects: the dispersion relation of polaritons with mass *M*, the gauge potential contribution of the RDSOC, the linear polarization splittings Δ and γ*k*^2^, and the energy relaxation mechanisms (*65*) via Λ, with zero energy level set at the bottom of the dispersion *E*_min_. *U* = *U*_D_ + *U*_R_ is the potential, where *U*_D_ is the disorder potential (correlation length, 7 μm; rms fluctuation, 0.3 meV) and $U_{R}=U_{0}\text{exp}\left(-r^{2}/2\sigma^{2}\right)$ is the repulsive potential of the reservoir. $\Gamma \left({|\psi |}^{2}\right)$ is the term combining decay and saturated gain, and *X* is the noise describing the spontaneous scattering from the excitonic reservoir. The saturated gain is given by $\Gamma \left({|\psi |}^{2}\right)=\Gamma_{0}\left(n_{R}\right)\text{exp}\left(-n_{\text{tot}}/n_{s}\right)$, where $n_{\text{tot}}=\int {|\psi |}^{2}\;\text{dxdy}$ is the total particle density, *n*_s_ is the saturation density, and *n*_R_ is the exciton reservoir density. Both the gain and the reservoir potential account for the duration of the pulsed excitation. *g*_1_ and *g*_2_ are the polariton-polariton interaction constants [same spin and opposite spin, respectively, with *g*_2_ < 0 and |*g*_2_| << *g*_1_ for negative detunings (*66*)]. We take *g*_2_ = 0, and *g*_1_ such as to have a maximal value of blue shift of 1 meV above threshold. We solve Eq. 9 numerically using the parameters of the polariton dispersion determined from the experimental results presented in Fig. 3. Other parameters were taken as Λ = 0.02, *U*_0_ = 0.1 meV, and σ = 18 μm. These parameters were used in simulations shown in Fig. 4.

A specificity of this platform with respect to atomic condensates is the mass difference γ between two linearly polarized modes coupled by the RDSOC. It ensures the presence of stripes even at zero detuning Δ with a contrast *M*/2*m* (see Eq. 3). However, stripes disappear at a particular nonzero value of detuning $\Delta =-\gamma k_{0}^{2}$.

## Supplementary Materials

### The PDF file includes:

Figs. S1 to S13 Supplementary Text Legends for movies S1 and S2 References
