# Supplementary material for: Observation of a supersolid phase in a spin-orbit coupled exciton-polariton Bose-Einstein condensate
Source: Sci Adv. 2026 Oct 16;12(42):eaee4875. doi: 10.1126/sciadv.aee4875 (PMC13674481; doi:10.1126/sciadv.aee4875)
Supplement: Supplementary file 1 [file sciadv.aee4875_sm.pdf]

Science Advances
AAAS

Supplementary Materials for

Observation of a supersolid phase in a spin-orbit coupled exciton-polariton Bose-Einstein condensate

Marcin Muszyński *et al.*

Corresponding author: Guillaume Malpuech, guillaume.malpuech@uca.fr;
Jacek Szczytko, jacek.szczytko@fuw.edu.pl

Sci. Adv. **12**, eaee4875 (2026)
DOI: 10.1126/sciadv.aee4875

The PDF file includes:

Figs. S1 to S13
Supplementary Text
Legends for movies S1 and S2
References

Other Supplementary Material for this manuscript includes the following:

Movies S1 and S2

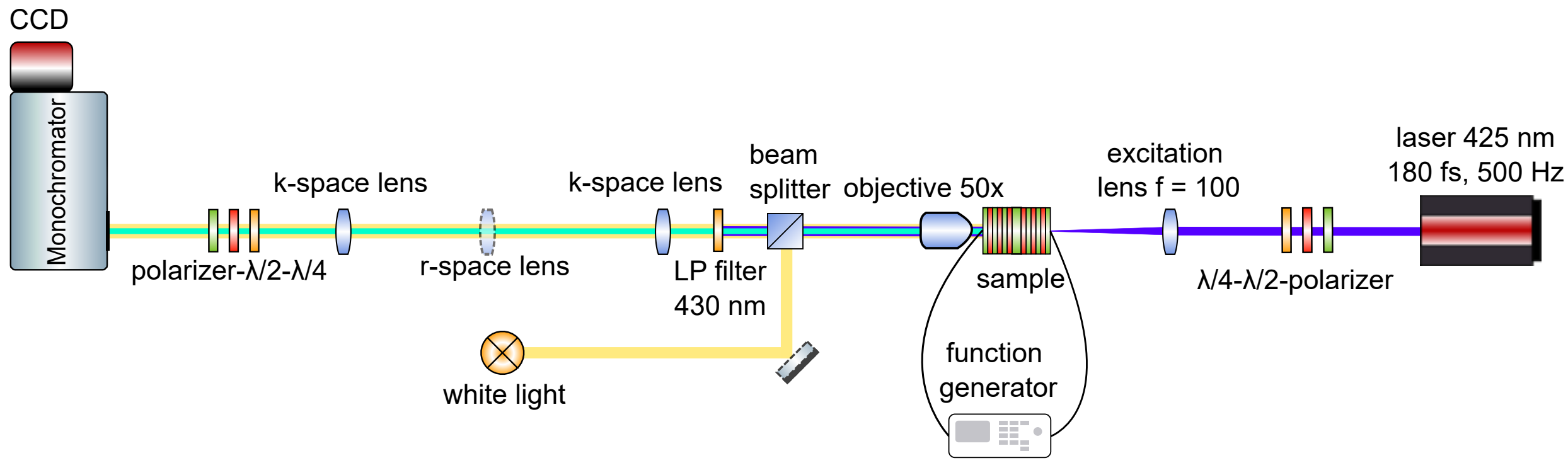

Figure S1: **Experimental setup scheme.**

Supplementary Text

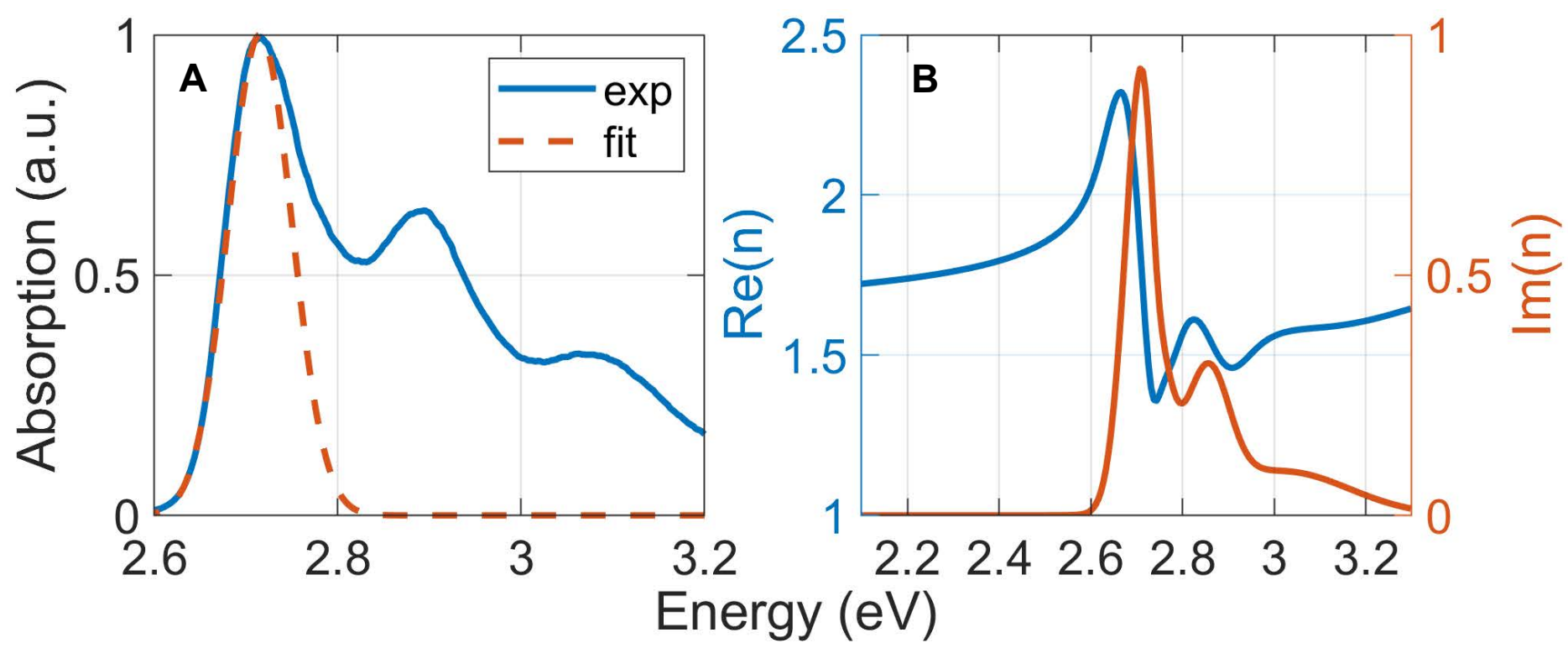

Figure S2: **MeLPPP absorption and complex refractive index.** (**A**): Experimentally measured absorption of a single MeLPPP layer on the DBR (solid blue line); the fit of the main absorption peak by Gaussian function representing inhomogeneous broadening of excitons (dashed red line); (**B**): real (Re(n), blue line) and imaginary (Im(n), red line) parts of complex refractive index obtained by variable angle spectroscopic ellipsometry.

Additional voltage for multiple-shot averaged experiment

In the main text, we have presented a set of results for sample "1", corresponding to a limited set of voltages. In order to confirm that the stripes can also be observed at other voltages in this sample, here we present an additional figure. Figure S6 shows the stripes observed in the total density at a different voltage (8.10 V) in the same set of experiments as in Fig. 3 of the main text, with averaging

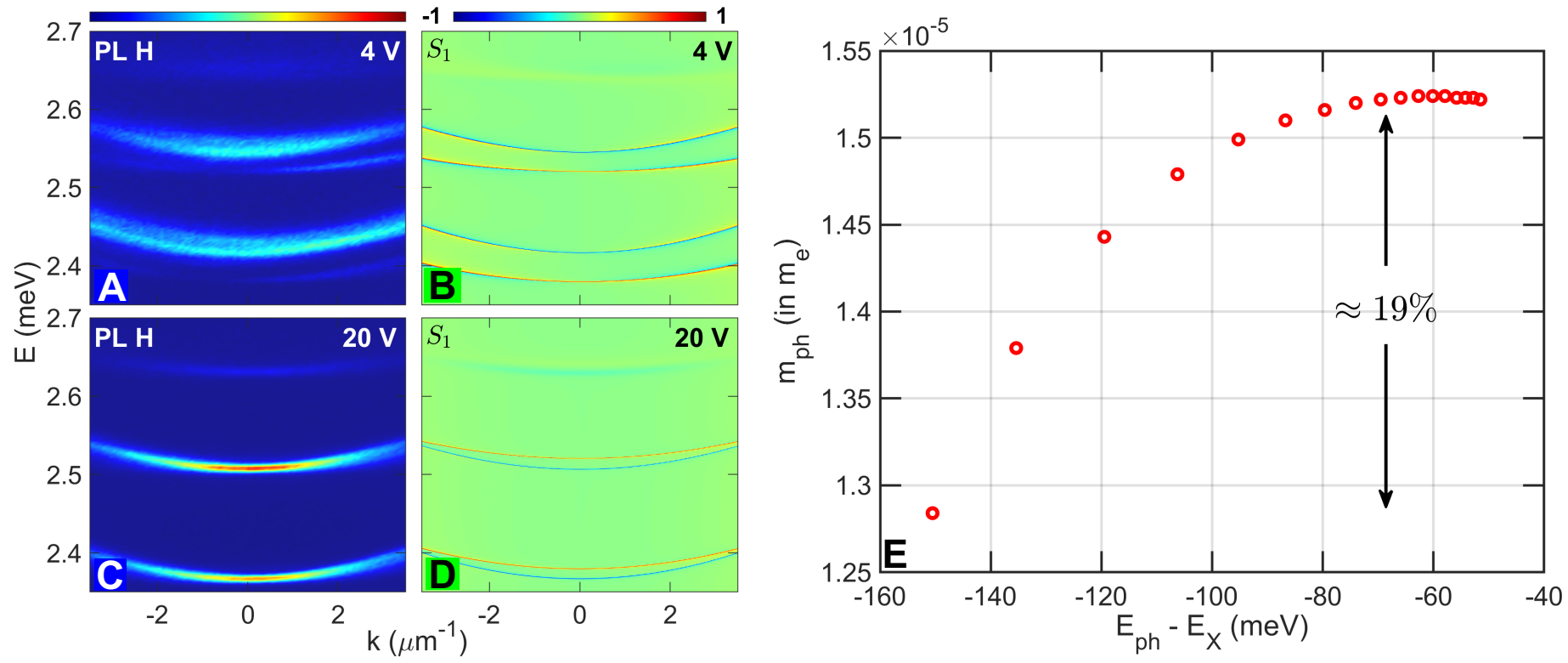

Figure S3: **Photonic mass extraction.** (**A**,**C**) Experimentally measured PL in H polarization and (**B**,**D**) numerically calculated first Stokes parameter (degree of linear polarization) with the use of the Berreman method for the voltage (A,B) 4 V and (C,D) 20 V applied to the microcavity. (**E**) Extracted from simulation, the photonic mass as a function of photonic mode detuning. The photonic detunings range corresponds to the LPB detunings range from Fig. 2(D). The total photonic mass change is 19%, proving that the mass change of 76% shown in Fig. 2(D) can be explained only by exciton-photon coupling.

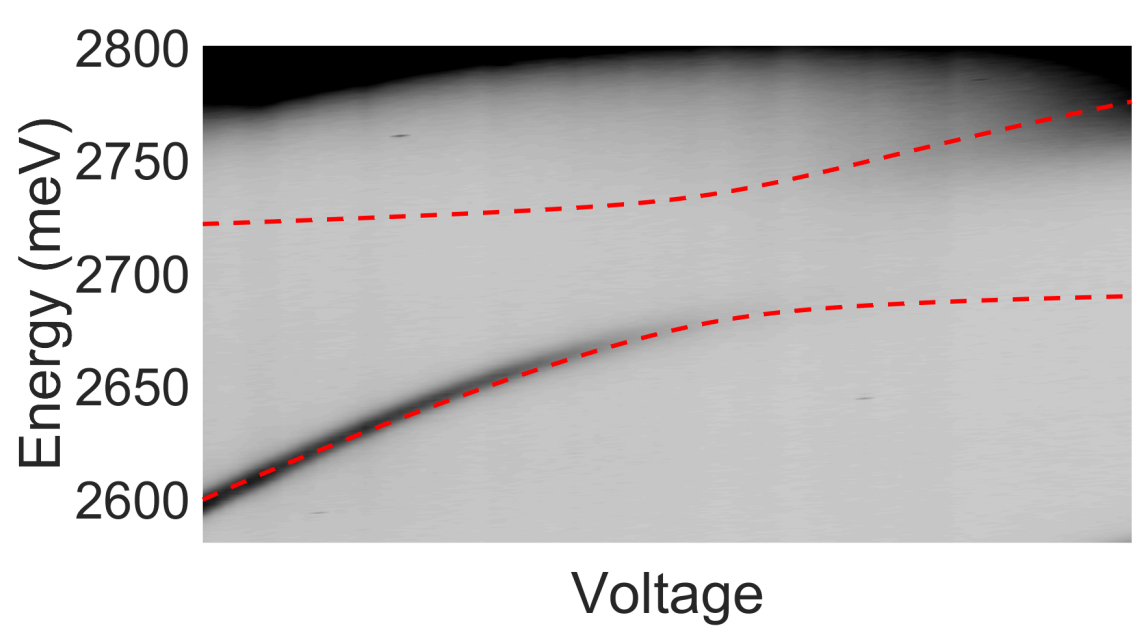

Figure S4: **Anticrossing of exciton-polariton modes.** White light transmission of the sample "3" versus the applied voltage. The dashed lines are computed from a two damped oscillator model as described in the text.

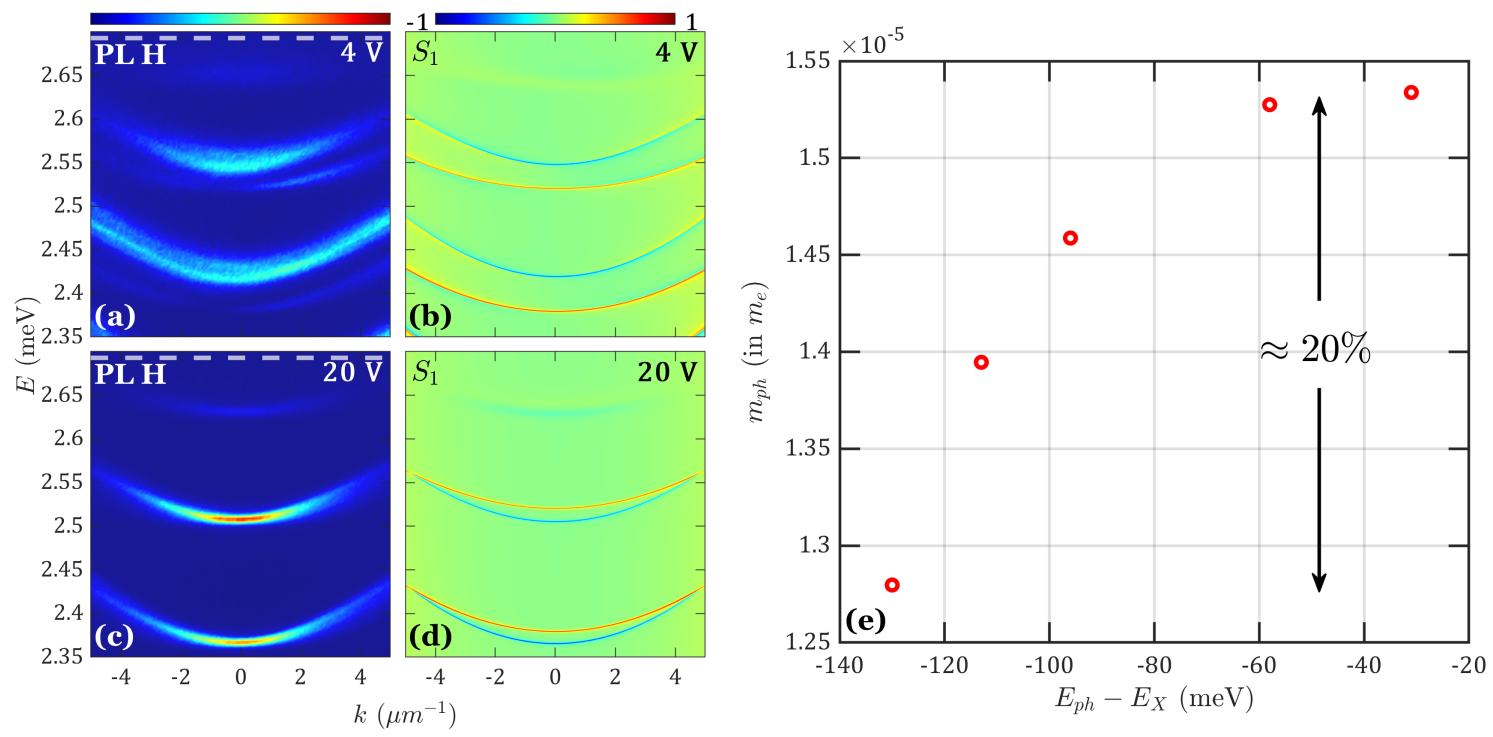

Figure S5: **Spatially extended coherence.** Experimental interferogram of a polariton condensate obtained by overlapping on the camera the original and inverted real-space image of the condensate emission going through the separate arms of Michelson interferometer at zero time-delay in a single-shot regime for sample 1. The interference fringes are visible over the whole area of the condensate showing long-range spatial coherence. Similar results were also obtained for sample 2.

over multiple pulses.

Experimental study of the condensate in the reciprocal space

The formation of a supersolid is a consequence of the condensation in a dispersion with two minima at non-zero wave vectors $\pm k_0$ (two degenerate ground states). The positions of the dispersion minima according to the effective Hamiltonian (see Eq. (1) in main text) are given by the following expression:

$$k_0 = \sqrt{\frac{\hbar^4\left(2\alpha^2+\gamma\Delta\right)-4M^2\gamma^2\left(2\alpha^2+\gamma\Delta\right)-2\sqrt{\hbar^4\alpha^2\left(\alpha^2+\gamma\Delta\right)\left(\hbar^4-4M^2\gamma^2\right)}}{4M^2\gamma^4-\gamma^2\hbar^4}} \tag{S1}$$

where $\Delta = E_V - E_H$ is the detuning between the H- and V-polarized modes, which is controlled by the applied voltage. An approximate expression neglecting γ is given in the main text (see Eq. 2). We have performed experiments above the condensation threshold, with the formation of a condensate in the two dispersion minima $\pm k_0$. An example of measured time-integrated emission intensity distribution as a function of wave vector k_x and energy is shown in Fig. 4 of the main text, with the intensity plotted as a false color in log scale, in order for the RDSOC dispersion to remain visible as compared to the bright emission of the condensate. We extract the difference between the two minima $\Delta k = 2k_0$ as a function of voltage. The results are plotted in Fig 4 of the main

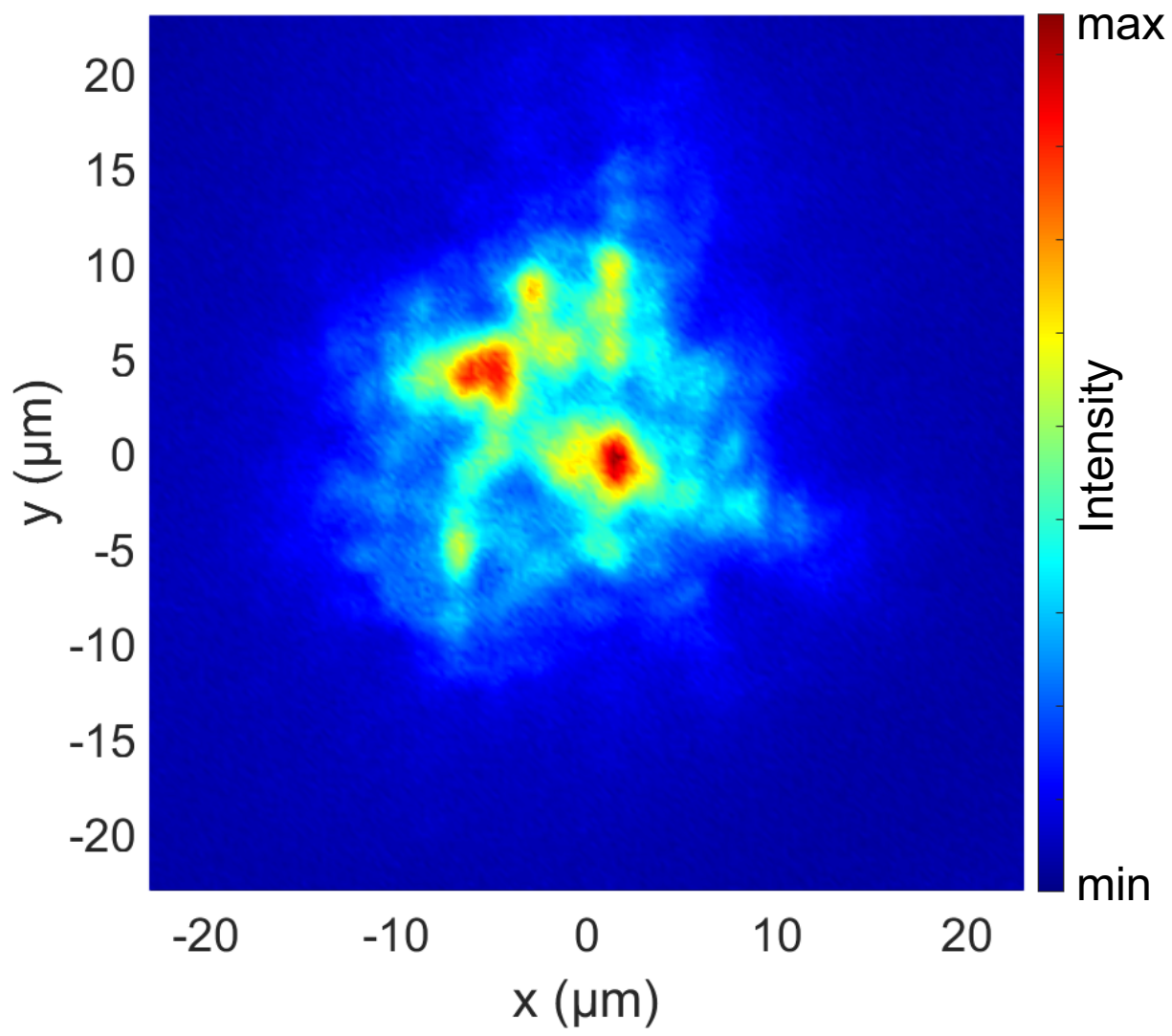

Figure S6: **Density stripes at a different voltage (sample "1").** Total intensity as a function of coordinates at 8.10 V.

text. The agreement of the experiment with the theory is very good, including both the square root dependence expected for the transition between one and two minima, and the asymmetry of the curve due to the parabolic H-V splitting γ.

Single shot stripes measured in total intensity

In this section, we present the experimental results obtained on sample "3". These results belong to the set of data used for Fig. 4 of the main text. Figure S7 shows two examples of single-shot images measured in total intensity. They demonstrate the formation of stripes in real space. The positions of these stripes are random, which is highlighted by the black dashed lines allowing to visualize the shift of the stripes between the two panels.

Contrast and statistics

The mathematical expectation of the contrast of persistent spin helix or density fringes is $C = 0$ for averaging over an infinite number of pulses. However, for a finite number of pulses, the contrast is never zero. The law according to which it tends to zero with the increase of the number of realizations can be found as follows. We consider that the intensity profile in each realization is

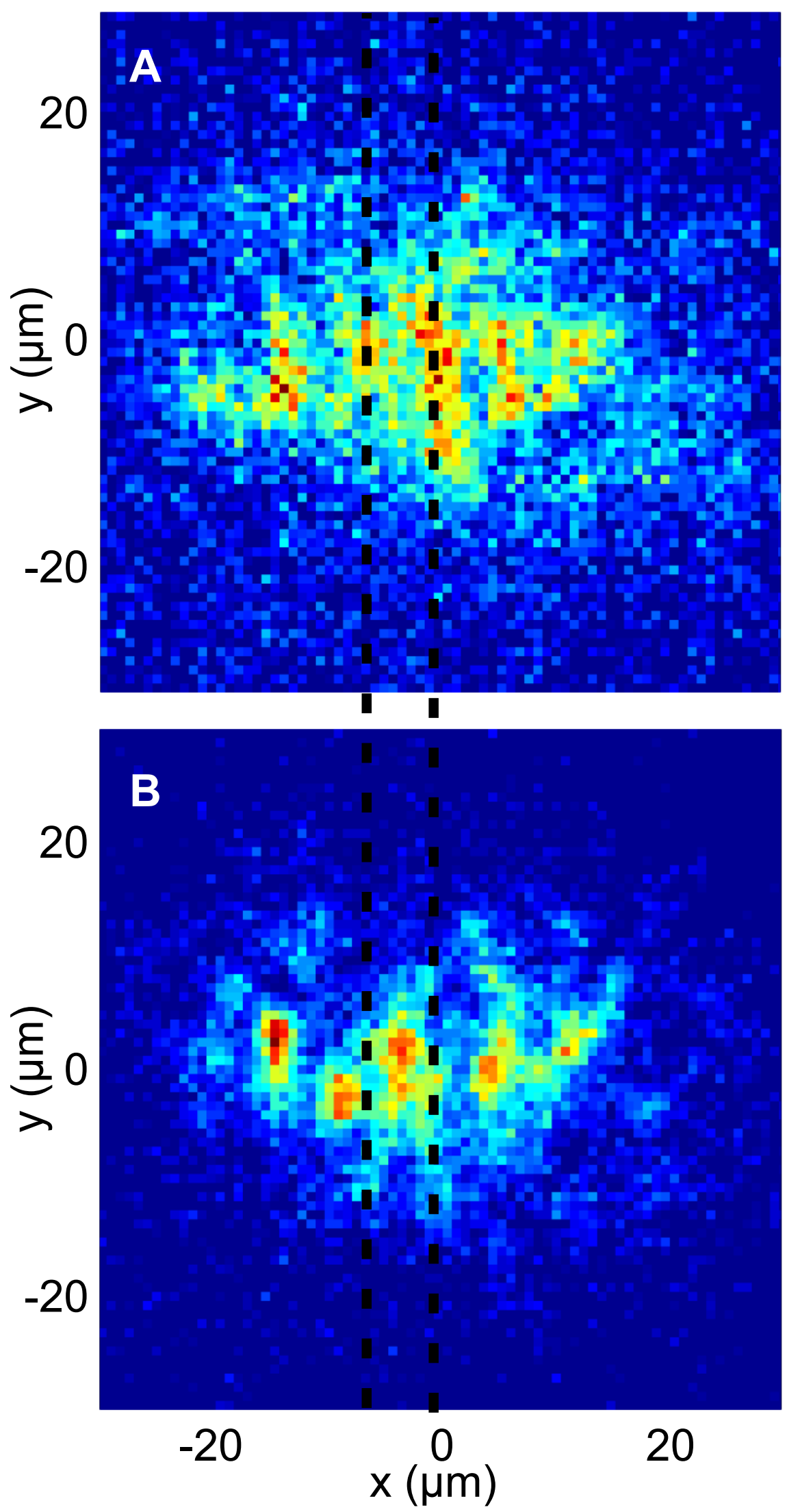

Figure S7: **Two single-shot images (A,B) showing the total intensity of emission from sample 3 exhibiting stripes.** Black dashed lines are the guides for the eyes allowing to visualize the shift of the stripes due to the spontaneous symmetry breaking.

given by $I_i(x) = \cos^2(kx + \eta_i)$, where η_i is a random phase due to spontaneous symmetry breaking. The total intensity for N realizations is given by

$$I(x) = \sum_{i=1}^{N} \cos^2(kx + \eta_i) \tag{S2}$$

Using trigonometric identities, we transform this to

$$I(x) = \frac{N}{2} + \frac{1}{2}\left(\cos 2kx \sum_{i=1}^{N} \cos 2\eta_i - \sin 2kx \sum_{i=1}^{N} \sin 2\eta_i\right) \tag{S3}$$

The two sums are correlated: they describe x and y coordinates of a random walker in 2D, which for large N gives $\sum_{i=1}^{N} \cos 2\eta_i \sim \sqrt{N} \cos 2\theta$, $\sum_{i=1}^{N} \sin 2\eta_i \sim \sqrt{N} \sin 2\theta$ where 2θ is a random direction from the origin towards the final position of the walker. This allows writing

$$I(x) = \frac{N}{2} + \frac{\sqrt{N}}{2} \cos(2kx + 2\theta) \tag{S4}$$

which gives the contrast

$$C(N) = \frac{I_{max} - I_{min}}{I_{max} + I_{min}} = \frac{\sqrt{N}}{N} = \frac{1}{\sqrt{N}} \tag{S5}$$

In Figure S8 we show an example of an intensity profile $I(y)$ for one given single-shot measurement (black) along a single line $x = const$, compared with the result of averaging (red) over all available single-shot images (over 500 for this experiment). No clear stripes are observed in the averaged case.

The decrease of the contrast calculated above can be seen as an application of the Berry-Esseen theorem (67,68) to describe how the sample distribution of the random walk tends to the normal distribution. This allows us to extend the consideration to the case of vortex formation via the Kibble-Zurek mechanism, leading to the appearance of dislocations in the interference pattern, which strongly reduces the contrast observed via the k-space intensity distribution, because of the broadening of the peaks. For random variables with non-identical distributions, the theorem states that the resulting distribution tends towards the normal distribution as $\left(\sum_{i=1}^{N} \sigma_i^2\right)^{-1/2}$. For identical σ, this gives the $1/\sqrt{N}$ law obtained above. However, the realizations containing vortices strongly differ from the realizations that do not contain one. The number of vortices in a given realization follows the Poisson distribution: $P_k = \lambda^k e^{-\lambda}/k!$ (the Kibble-Zurek mechanism predicts the average density of vortices for a given set of parameters, and the Poissonian distribution predicts their number in a

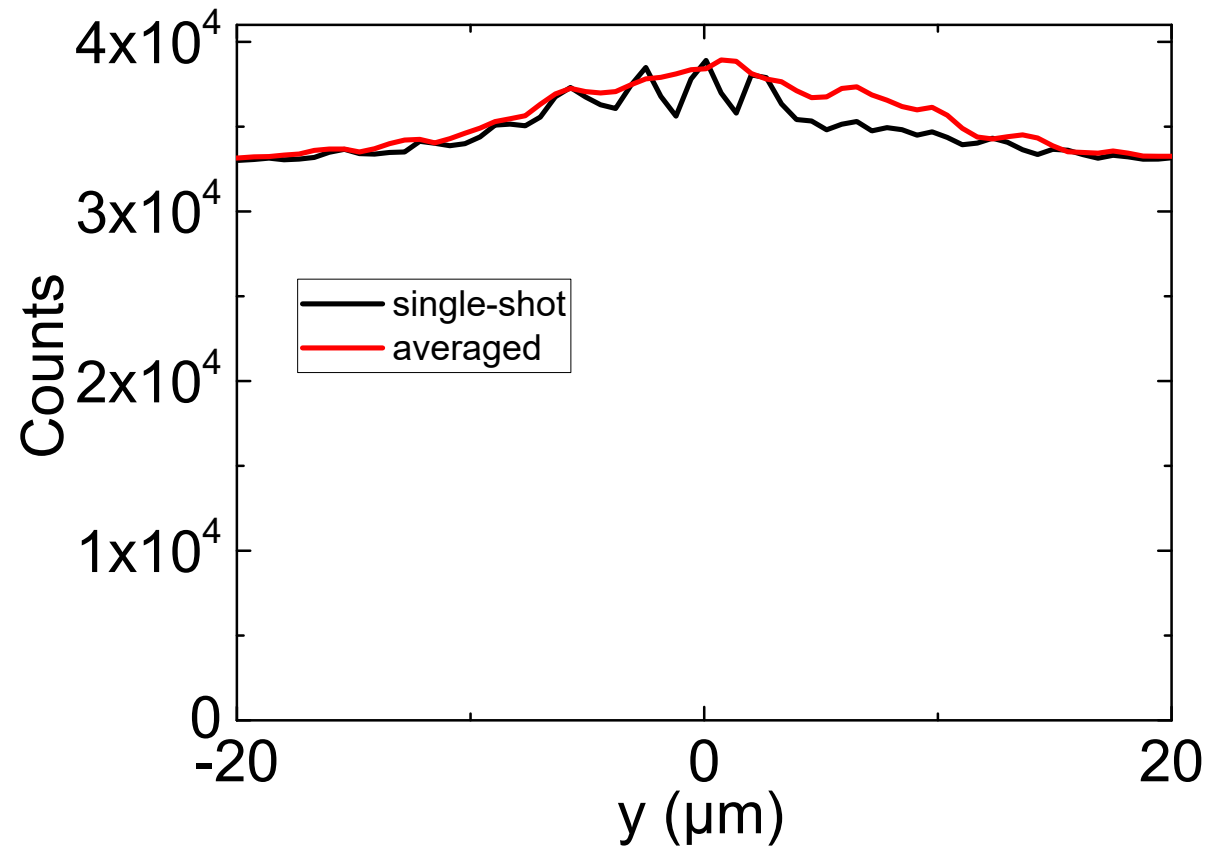

Figure S8: **Comparison of single-shot and integrated intensities.** The intensity $I(y)$ for one single-shot measurement (black) and for the averaged case (red), where the contrast is smeared out due to spontaneous symmetry breaking.

finite-size system corresponding to this density). We assume that the number of vortices is small: $\lambda \sim 1$, and simplify the consideration by assuming at most 1 vortex in the system. We can therefore group the terms as

$$\frac{1}{\sqrt{\sum_{i=1}^{N} \sigma_i^2}} = \frac{1}{\sqrt{n_0 \sigma_0^2 + n_1 \sigma_1^2}} \tag{S6}$$

with $n_0 + n_1 = N$ (n_0 is the number of realizations with zero vortices, n_1 with 1 vortex). At high N, the number of vortices converges to the Poisson distribution, and we can write $n_1 = NP_1 \approx N/e$, in which case one can factorize by $\sqrt{N}$ and obtain

$$C \sim \frac{1}{\sqrt{N}\sqrt{\sigma_0^2 + e^{-1}\sigma_1^2}} \tag{S7}$$

meaning that the $N^{-1/2}$ power law is recovered at large N even in presence of vortices. The cases with more than 1 vortex allowed can be treated similarly, as soon as their total number is proportional to N according to the Poisson distribution.

However, for a finite realization set one can expect a deviation from the average number of vortices. This deviation between the set distribution and an ideal probability distribution is itself described by the Berry-Esseen theorem. The number of realizations with a single vortex can

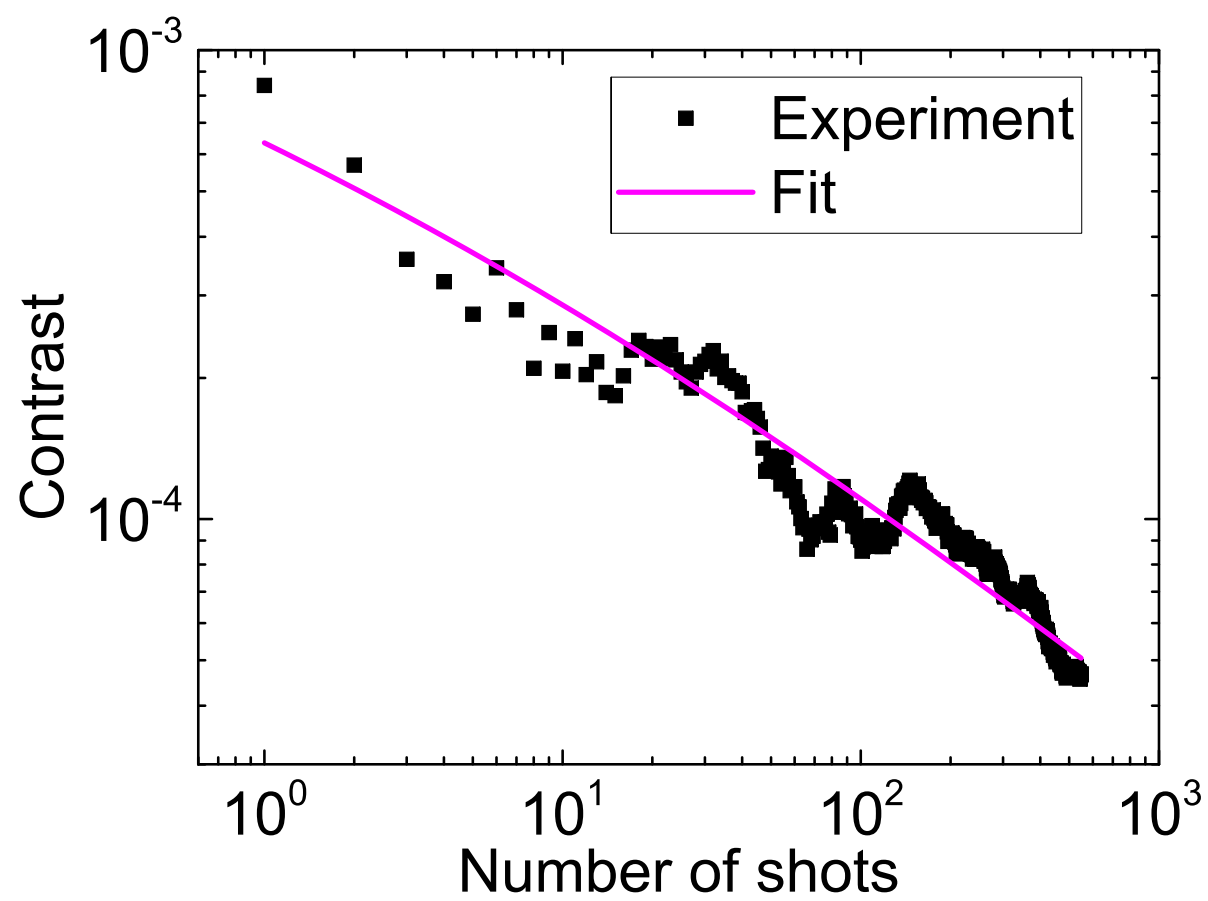

Figure S9: **Contrast decrease with the number of shots.** The experimentally-measured contrast as a function of number of averaged single shots and its theoretical fit based on Eq. (S9).

therefore be expected to behave as

$$n_1 \sim \left(1 + A/\sqrt{N}\right) NP_1 \tag{S8}$$

The overall expression for the contrast for a finite number of realizations can be written as

$$C \sim \frac{A}{\sqrt{N + B\sqrt{N}}} \tag{S9}$$

where A and B are fitting parameters. Qualitatively, the power law decrease of the contrast appears slower for small N and tends to $N^{-1/2}$ for large N. The transition between the two regimes is determined by $NP_1 \sim 1$ (that is, when there is on average at least one vortex in N realizations). This function is used in Fig. S9 showing the same data as in Fig. 4 of the main text (sample "2").

We have checked the validity of this model by comparing it with additional large scale numerical simulations (up to $N = 10^5$, with additional averaging) involving periodic stripes with random vortices with a Poissonian distribution.

Interaction mechanisms

We stress that in order to explain the superfluidity leading to the motion of the stripes, the interactions should be of the order of 1-2 meVs, comparable with the disorder magnitude. Direct exciton-exciton interaction is small in organic materials and also in large band gap semiconductors. For these materials, polariton-polariton interactions are mediated by the screening of the exciton

oscillator strength by carrier phase space filling, which provides interaction energies proportional to the Rabi splitting, which is huge for these materials. The magnitude of the effect and its efficiency were recently demonstrated in GaN-based structures, where direct exciton-exciton interactions are certainly weak, but where a polariton mode-locked laser has been reported (69), clearly demonstrating polariton-polariton interaction in the meV range.

Bogolon dispersion of the supersolid

We have performed additional numerical simulations in order to demonstrate that the condensate formed in presence of the RDSOC can, at least in principle, exhibit superfluid properties. We use the same Gross-Pitaevskii equation as in Eq. (9) from main text (Methods). However, we focus on an idealized case of a homogeneous infinite system that we simulate by using periodic boundary conditions and a homogeneous pump, with the particle density corresponding to a blue shift of 0.25 meV. We also add a small propagating potential defect $U(x,y,t) = U_{def}\exp\left(-(x-vt-x_0)^2/2\sigma_d^2\right)\exp\left(-y^2/2\sigma_d^2\right)$ instead of the reservoir potential (which we choose as the zero energy reference because it is now homogeneous). Here, $U_{def} = 3.5$ meV is the amplitude of the defect potential, $\sigma_d = 2.8$ μm is the size of the defect, $(x_0, 0)$ is its initial position in the XY plane, v is its velocity. We plot the Fourier transform of the solution (the probability density as a function of the momentum and energy) in Fig. S10.

Panel (A) shows the bogolon dispersion without a moving defect (here we take the interaction constant $g_2 = -0.2g_1$ to demonstrate two different sound velocities due to spin-anisotropic interactions). Panel (B) shows the subsonic propagation case ($v = 0.3$ μm/ps, while the lowest speed of sound is $c \approx 1.1$ μm/ps). The defect perturbs the density profile of the condensate. This localized perturbation appears in the Fourier transform as a tilted straight line (with the angle corresponding to the velocity of the defect) crossing the condensate. If this line does not cross the bogolon dispersion, the scattering on the defect cannot perturb the condensate and there is no friction or viscosity. This configuration (panel B) corresponds to the Landau criterion of superfluidity in the reference frame of immobile condensate and propagating defect. Panel (C) shows a case with both a moving defect and a static disorder potential, corresponding to the disorder observed experimentally with an optical microscope (Fig. S11). The superfluid density adjusts itself to the disorder potential profile and the line appearing horizontal is the Fourier transform of the modulated superfluid density. Panel

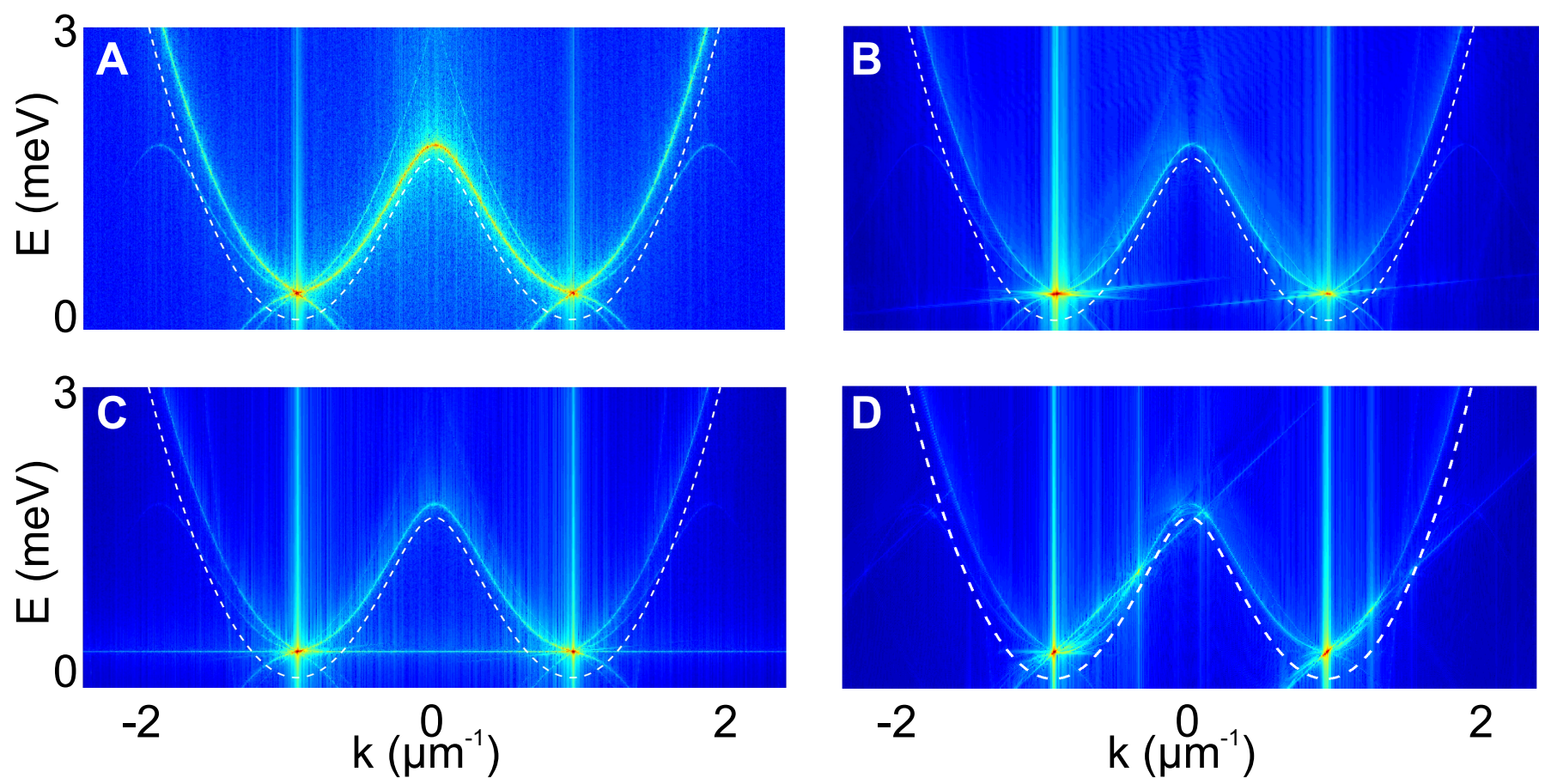

Figure S10: **Superfluid and supersonic regimes in a supersolid.** Probability density as a function of energy and wave vector $|\psi(k_x, E)|^2$: (**A**) Bogoliubov dispersion with two different sound velocities visible due to spin-anisotropic interactions; (**B**) subsonic defect propagation at velocity $v < c$, where c is the speed of sound, no resonant scattering from the condensate; (**C**) subsonic defect propagation $v < c$ in presence of a stationary disorder potential, no resonant scattering from the condensate: the condensate is still superfluid; (**D**) supersonic defect propagation $v > c$, visible resonant scattering from the condensate.

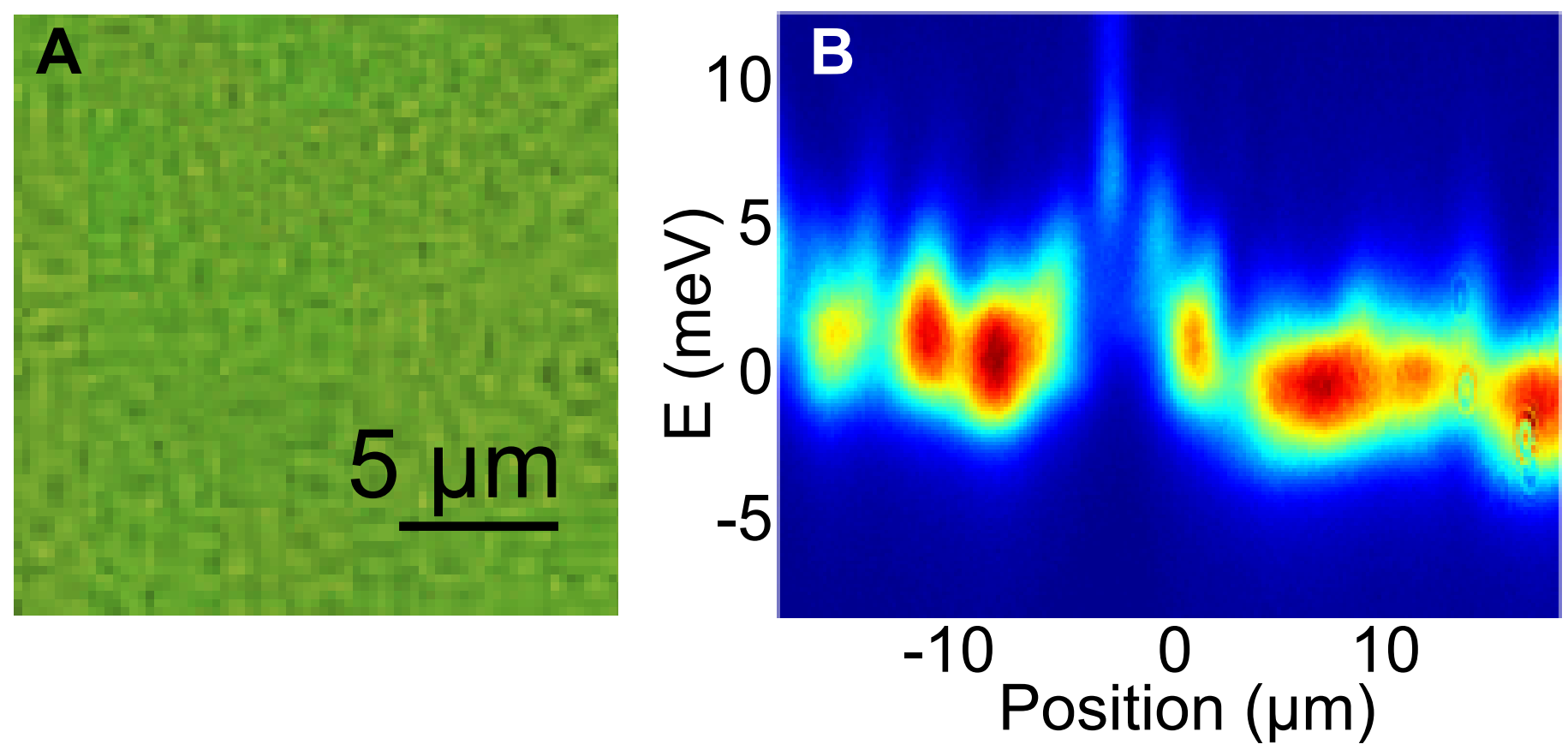

Figure S11: **Experimental measurement of the photonic disorder.** (**A**) An image of a sample with MeLPPP obtained with an optical microscope, demonstrating a visible presence of disorder. (**B**) Spatially-resolved spectrum of polariton modes demonstrating the variation of energy on a few meV scale with micrometer correlation length.

(D) is with no disorder, but with a defect moving faster than the speed of sound. The tilted straight line corresponds to the velocity of the defect $v = 2$ μm/ps and crosses the dispersion of bogolons. Resonant (in the frame moving with the defect) scattering from the condensate to the bogolon states can be observed in this case, which corresponds to losses due to friction. Panels (B,C) therefore demonstrate the possibility of superfluid defect motion in a supersolid, as compared with panel (D). We note that in systems with spatial periodicity, the scattering from the condensate can occur towards higher-order Brillouin zones. These are the so-called Umklapp processes (53,54). To avoid them, the defect needs to be sufficiently smooth.

Movies of numerical simulations

In this section, we present additional Supplementary Movies.

We present the results of realistic numerical simulations corresponding to the second movie (the one with a disorder potential) in Fig. S12. These simulations, based on the same Gross-Pitaevskii equation as above (Eq. (9) from main text) include pulsed pumping and disorder. The two panels of the figure show that the condensate indeed forms in the two minima of the dispersion at $\pm k_0$ and that the time-averaged intensity exhibits stripes, corresponding to the formation of a supersolid.

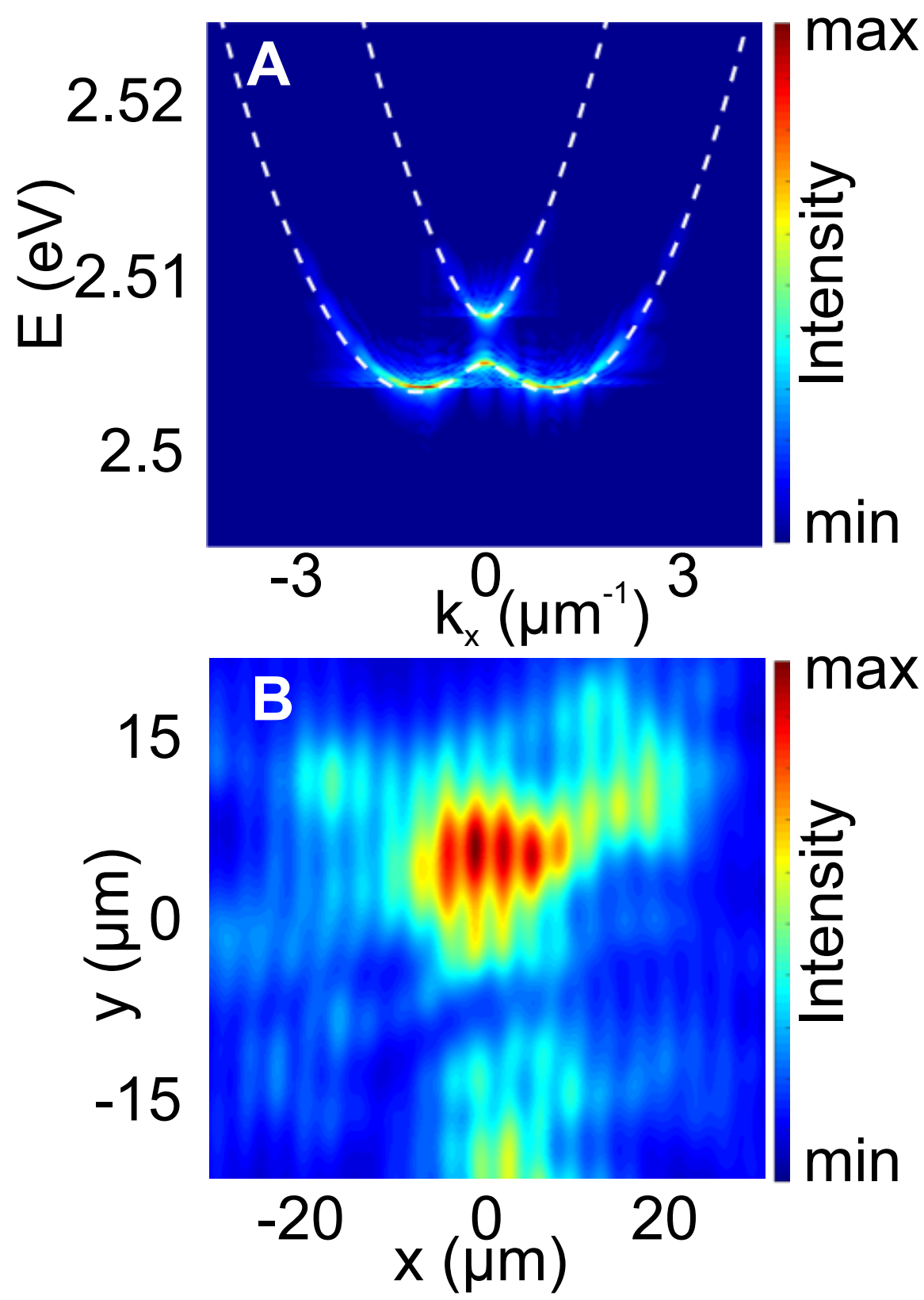

Figure S12: **Realistic numerical simulations.** The time-averaged intensity distribution (**A**) as a function of wave vector and energy (the bare polariton dispersion is plotted as a dashed line) and (**B**) in real space, exhibiting clear stripes.

The parameters of these simulations, corresponding to Fig. 3 of the main text, are similar to those of Fig. 4, except the duration of the pulsed excitation, which is short in this case. The time dependencies of the gain and the reservoir potential account for the decay and depletion of the reservoir (see Ref. 45 of the main text).

Thermal distribution function

To demonstrate the thermalization of our system and the quasi-equilibrium nature of our condensate, we extract the occupation numbers of the polariton states and show that they are well described with a Bose-Einstein distribution function.

Our system is two-dimensional, and at sufficiently large energies its dispersion corresponds to two parabolas, so the density of states is constant, which allows to use the angle-integrated emission

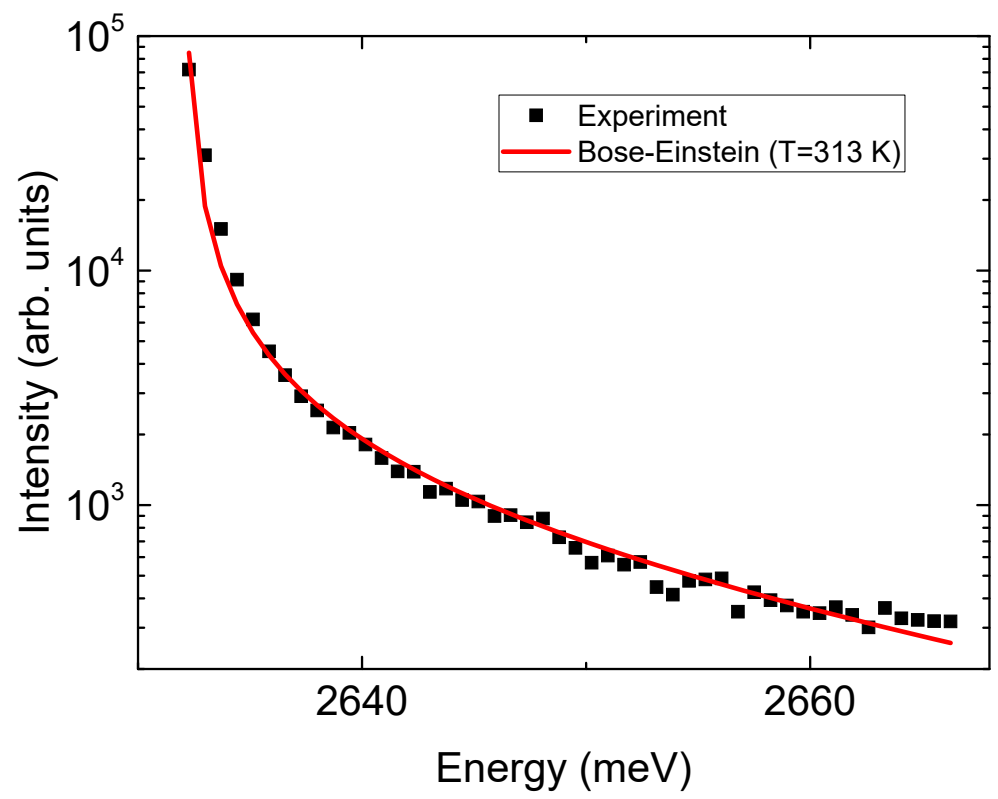

Figure S13: **Thermal distribution of the polariton population.** Angular-integrated intensity of polariton emission (from Fig. 4(A) of the main text) as a function of energy (black dots) together with a fit with a Bose-Einstein distribution function with a temperature $T = 313$ K (red solid line).

intensity as a good indication of the distribution function. The detuning is also not varying much within the considered energy range. We plot the angle-integrated emission intensity (corresponding to Fig. 4(A) of the main text) as a function of energy in Fig. S13 with black dots. The data is plotted in semi-log scale. In this representation, the exponential Boltzmann tail of the distribution function should show up as a straight line, which is indeed the case. We fit the experimental occupancies with a Bose-Einstein distribution function. The result is shown as a solid red curve in Fig. S13. The extracted temperature is $T = 313 \pm 45$ K. The high quality of the fit $R^2 = 0.987$ confirms that the system is indeed relatively close to thermal equilibrium and that the transition can be seen as a thermodynamic Bose-Einstein condensation.

Caption for Movie S1. Simulation with a vortex. The movie presents the numerically calculated polariton density as a function of time without disorder potential. A vortex appears during the condensation via the Kibble-Zurek mechanism, it is clearly visible as a dislocation of the stripes pattern in the middle of the figure.

Caption for Movie S2. Simulation with disorder. The movie presents the numerically calculated polariton density as a function of time in presence of disorder.

REFERENCES

1. A. J. Leggett, Can a solid be “superfluid”? *Phys. Rev. Lett.* **25**, 1543–1546 (1970).

2. M. H.-W. Chan, R. Hallock, L. Reatto, Overview on solid ^{4}He and the issue of supersolidity. *J. Low Temp. Phys.* **172**, 317–363 (2013).

3. E. Poli, T. Bland, S. J. M. White, M. J. Mark, F. Ferlaino, S. Trabucco, M. Mannarelli, Glitches in rotating supersolids. *Phys. Rev. Lett.* **131**, 223401 (2023).

4. J. Léonard, A. Morales, P. Zupancic, T. Esslinger, T. Donner, Supersolid formation in a quantum gas breaking a continuous translational symmetry. *Nature* **543**, 87–90 (2017).

5. J. D. Koralek, C. P. Weber, J. Orenstein, B. A. Bernevig, S.-C. Zhang, S. Mack, D. Awschalom, Emergence of the persistent spin helix in semiconductor quantum wells. *Nature* **458**, 610–613 (2009).

6. Y.-J. Lin, K. Jiménez-García, I. B. Spielman, Spin–orbit-coupled Bose–Einstein condensates. *Nature* **471**, 83–86 (2011).

7. J.-R. Li, J. Lee, W. Huang, S. Burchesky, B. Shteynas, F. Ç. Top, A. O. Jamison, W. Ketterle, A stripe phase with supersolid properties in spin–orbit-coupled Bose–Einstein condensates. *Nature* **543**, 91–94 (2017).

8. K. T. Geier, G. I. Martone, P. Hauke, W. Ketterle, S. Stringari, Dynamics of stripe patterns in supersolid spin-orbit-coupled bose gases. *Phys. Rev. Lett.* **130**, 156001 (2023).

9. C. Chisholm, S. Hirthe, V. Makhalov, R. Ramos, R. Vatré, J. Cabedo, A. Celi, L. Tarruell, Probing supersolidity through excitations in a spin-orbit–coupled Bose-Einstein condensate. *Science* **391**, 480–484 (2026).

10. L. Tanzi, J. Maloberti, G. Biagioni, A. Fioretti, C. Gabbanini, G. Modugno, Evidence of superfluidity in a dipolar supersolid from nonclassical rotational inertia. *Science* **371**, 1162–1165 (2021).

11. G. Biagioni, N. Antolini, B. Donelli, L. Pezzè, A. Smerzi, M. Fattori, A. Fioretti, C. Gabbanini, M. Inguscio, L. Tanzi, G. Modugno, Measurement of the superfluid fraction of a supersolid by Josephson effect. *Nature* **629**, 773–777 (2024).

12. E. Casotti, E. Poli, L. Klaus, A. Litvinov, C. Ulm, C. Politi, M. J. Mark, T. Bland, F. Ferlaino, Observation of vortices in a dipolar supersolid. *Nature* **635**, 327–331 (2024).

13. M. Matuszewski, T. Taylor, A. V. Kavokin, Exciton supersolidity in hybrid Bose-Fermi systems. *Phys. Rev. Lett.* **108**, 060401 (2012).

14. I. Carusotto, C. Ciuti, Quantum fluids of light. *Rev. Mod. Phys.* **85**, 299–366 (2013).

15. A. V. Kavokin, J. J. Baumberg, G. Malpuech, F. P. Laussy, *Microcavities*, vol. 21 (Oxford Univ. Press, 2017).

16. A. Amo, J. Lefrère, S. Pigeon, C. Adrados, C. Ciuti, I. Carusotto, R. Houdré, E. Giacobino, A. Bramati, Superfluidity of polaritons in semiconductor microcavities. *Nat. Phys.* **5**, 805–810 (2009).

17. G. Lerario, A. Fieramosca, F. Barachati, D. Ballarini, K. S. Daskalakis, L. Dominici, M. De Giorgi, S. A. Maier, G. Gigli, S. Kéna-Cohen, D. Sanvitto, Room-temperature superfluidity in a polariton condensate. *Nat. Phys.* **13**, 837–841 (2017).

18. D. Caputo, D. Ballarini, G. Dagvadorj, C. Sánchez Muñoz, M. De Giorgi, L. Dominici, K. West, L. N. Pfeiffer, G. Gigli, F. P. Laussy, M. H. Szymanska, D. Sanvitto, Topological order and thermal equilibrium in polariton condensates. *Nat. Mater.* **17**, 145–151 (2018).

19. D. Trypogeorgos, A. Gianfrate, M. Landini, D. Nigro, D. Gerace, I. Carusotto, F. Riminucci, K. W. Baldwin, L. N. Pfeiffer, G. I. Martone, M. De Giorgi, D. Ballarini, D. Sanvitto, Emerging supersolidity in photonic-crystal polariton condensates. *Nature* **639**, 337–341 (2025).

20. Y. Meng, W. Li, K. Peng, C. Ti, J. Dang, X. Wu, X. Han, W. Bao, Hybrid perovskite–nanograting photonic architecture enables supersolidity at room temperature. *Nat. Nanotechnol.* **21**, 663–671 (2026).

21. X. Zhai, C. Xing, X. Yang, X. Zhang, H. Dai, X. Wang, A. Pan, S. Schumacher, X. Ma, T. Gao, Electrically tunable nonrigid moire exciton polariton supersolids at room temperature. arXiv:2504.11057 [physics.optics] (2025).

22. V. Ardizzone, F. Riminucci, S. Zanotti, A. Gianfrate, M. Efthymiou-Tsironi, D. Suàrez-Forero, F. Todisco, M. De Giorgi, D. Trypogeorgos, G. Gigli, K. Baldwin, L. Pfeiffer, D. Ballarini, H. S. Nguyen, D. Gerace, D. Sanvitto, Polariton Bose–Einstein condensate from a bound state in the continuum. *Nature* **605**, 447–452 (2022).

23. F. Riminucci, A. Gianfrate, D. Nigro, V. Ardizzone, S. Dhuey, L. Francaviglia, K. Baldwin, L. Pfeiffer, D. Ballarini, D. Trypogeorgos, A. Schwartzberg, D. Gerace, D. Sanvitto, Polariton condensation in gap-confined states of photonic crystal waveguides. *Phys. Rev. Lett.* **131**, 246901 (2023).

24. I. Septembre, I. Foudjo, V. Develay, T. Guillet, S. Bouchoule, J. Zúñiga-Pérez, D. D. Solnyshkov, G. Malpuech, Soliton formation in an exciton-polariton condensate at a bound state in the continuum. *Phys. Rev. B* **109**, 205302 (2024).

25. A. Kavokin, G. Malpuech, M. Glazov, Optical spin Hall effect. *Phys. Rev. Lett.* **95**, 136601 (2005).

26. C. Leyder, M. Romanelli, J. P. Karr, E. Giacobino, T. C. H. Liew, M. M. Glazov, A. V. Kavokin, G. Malpuech, A. Bramati, Observation of the optical spin Hall effect. *Nat. Phys.* **3**, 628–631 (2007).

27. H. Terças, H. Flayac, D. Solnyshkov, G. Malpuech, Non-Abelian gauge fields in photonic cavities and photonic superfluids. *Phys. Rev. Lett.* **112**, 066402 (2014).

28. A. Nalitov, D. Solnyshkov, G. Malpuech, Polariton Z topological insulator. *Phys. Rev. Lett.* **114**, 116401 (2015).

29. S. Klembt, T. Harder, O. Egorov, K. Winkler, R. Ge, M. Bandres, M. Emmerling, L. Worschech, T. Liew, M. Segev, C. Schneider, S. Hofling, Exciton-polariton topological insulator. *Nature* **562**, 552–556 (2018).

30. S. Richter, H.-G. Zirnstein, J. Zúñiga-Pérez, E. Krüger, C. Deparis, L. Trefflich, C. Sturm, B. Rosenow, M. Grundmann, R. Schmidt-Grund, Voigt exceptional points in an anisotropic ZnO-based planar microcavity: Square-root topology, polarization vortices, and circularity. *Phys. Rev. Lett.* **123**, 227401 (2019).

31. K. Rechcińska, M. Król, R. Mazur, P. Morawiak, R. Mirek, K. Łempicka, W. Bardyszewski, M. Matuszewski, P. Kula, W. Piecek, P. G. Lagoudakis, B. PiĘtka, J. Szczytko, Engineering spin-orbit synthetic Hamiltonians in liquid-crystal optical cavities. *Science* **366**, 727–730 (2019).

32. A. Gianfrate, O. Bleu, L. Dominici, V. Ardizzone, M. De Giorgi, D. Ballarini, G. Lerario, K. W. West, L. N. Pfeiffer, D. D. Solnyshkov, D. Sanvitto, G. Malpuech, Measurement of the quantum geometric tensor and of the anomalous Hall drift. *Nature* **578**, 381–385 (2020).

33. J. Ren, Q. Liao, F. Li, Y. Li, O. Bleu, G. Malpuech, J. Yao, H. Fu, D. Solnyshkov, Nontrivial band geometry in an optically active system. *Nat. Commun.* **12**, 689 (2021).

34. M. Król, H. Sigurdsson, K. Rechcińska, P. Oliwa, K. Tyszka, W. Bardyszewski, A. Opala, M. Matuszewski, P. Morawiak, R. Mazur, W. Piecek, P. Kula, P. G. Lagoudakis, B. Pietka, J. Szczytko, Observation of second-order meron polarization textures in optical microcavities. *Optica* **8**, 255–261 (2021).

35. L. Polimeno, G. Lerario, M. De Giorgi, L. De Marco, L. Dominici, F. Todisco, A. Coriolano, V. Ardizzone, M. Pugliese, C. T. Prontera, V. Maiorano, A. Moliterni, C. Giannini, V. Olieric, G. Gigli, D. Ballarini, Q. Xiong, A. Fieramosca, D. D. Solnyshkov, G. Malpuech, D. Sanvitto, Tuning of the Berry curvature in 2D perovskite polaritons. *Nat. Nanotechnol.* **16**, 1349–1354 (2021).

36. M. S. Spencer, Y. Fu, A. P. Schlaus, D. Hwang, Y. Dai, M. D. Smith, D. R. Gamelin, X.-Y. Zhu, Spin-orbit–coupled exciton-polariton condensates in lead halide perovskites. *Sci. Adv.* **7**, eabj7667 (2021).

37. T. Long, X. Ma, J. Ren, F. Li, Q. Liao, S. Schumacher, G. Malpuech, D. Solnyshkov, H. Fu, Helical polariton lasing from topological valleys in an organic crystalline microcavity. *Adv. Sci.* **9**, e2203588 (2022).

38. M. Król, I. Septembre, P. Oliwa, , Annihilation of exceptional points from different Dirac valleys in a 2D photonic system. *Nat. Commun.* **13**, 5340 (2022).

39. J. Liang, W. Wen, F. Jin, Y. G. Rubo, T. C. Liew, R. Su, Polariton spin Hall effect in a Rashba–Dresselhaus regime at room temperature. *Nat. Photonics* **18**, 357–362 (2024).

40. K. Y. Bliokh, A. Niv, V. Kleiner, E. Hasman, Geometrodynamics of spinning light. *Nat. Photonics* **2**, 748–753 (2008).

41. K. Łempicka-Mirek, M. Król, H. Sigurdsson, A. Wincukiewicz, P. Morawiak, R. Mazur, M. Muszyński, W. Piecek, P. Kula, T. Stefaniuk, M. Kamińska, L. De Marco, P. G. Lagoudakis, D. Ballarini, D. Sanvitto, J. Szczytko, B. Piętka, Electrically tunable Berry curvature and strong light-matter coupling in liquid crystal microcavities with 2D perovskite. *Sci. Adv.* **8**, eabq7533 (2022).

42. Y. Li, X. Ma, X. Zhai, M. Gao, H. Dai, S. Schumacher, T. Gao, Manipulating polariton condensates by Rashba-Dresselhaus coupling at room temperature. *Nat. Commun.* **13**, 3785 (2022).

43. K. Łempicka Mirek, M. Król, L. D. Marco, A. Coriolano, L. Polimeno, I. Viola, M. Kędziora, M. Muszyński, P. Morawiak, R. Mazur, P. Kula, W. Piecek, P. Fita, D. Sanvitto, J. Szczytko, B. Piętka, Electrical polarization switching of perovskite polariton laser. *Nanophotonics* **13**, 2659–2668 (2024).

44. U. Scherf, A. Bohnen, K. Müllen, Polyarylenes and poly(arylenevinylene)s, 9. The oxidized states of a (1,4-phenylene) ladder polymer. *Makromol. Chem.* **193**, 1127–1133 (1992).

45. J. D. Plumhof, T. Stöferle, L. Mai, U. Scherf, R. F. Mahrt, Room-temperature Bose–Einstein condensation of cavity exciton–polaritons in a polymer. *Nat. Mater.* **13**, 247–252 (2014).

46. A. V. Zasedatelev, A. V. Baranikov, D. Sannikov, D. Urbonas, F. Scafirimuto, V. Y. Shishkov, E. S. Andrianov, Y. E. Lozovik, U. Scherf, T. Stöferle, R. F. Mahrt, P. G. Lagoudakis, Single-photon nonlinearity at room temperature. *Nature* **597**, 493–497 (2021).

47. A. V. Zasedatelev, A. V. Baranikov, D. Urbonas, F. Scafirimuto, U. Scherf, T. Stöferle, R. F. Mahrt, P. G. Lagoudakis, A room-temperature organic polariton transistor. *Nat. Photonics* **13**, 378–383 (2019).

48. F. Scafirimuto, D. Urbonas, U. Scherf, R. Mahrt, T. Stöferle, Room-temperature exciton-polariton condensation in a tunable zero-dimensional microcavity. *ACS Photonics* **5**, 85–89 (2018).

49. R. Onofrio, C. Raman, J. Vogels, J. Abo-Shaeer, A. Chikkatur, W. Ketterle, Observation of superfluid flow in a Bose-Einstein condensed gas. *Phys. Rev. Lett.* **85**, 2228–2231 (2000).

50. A. Chikkatur, A. Görlitz, D. Stamper-Kurn, S. Inouye, S. Gupta, W. Ketterle, Suppression and enhancement of impurity scattering in a Bose-Einstein condensate. *Phys. Rev. Lett.* **85**, 483–486 (2000).

51. E. Wertz, A. Amo, D. D. Solnyshkov, L. Ferrier, T. C. H. Liew, D. Sanvitto, P. Senellart, I. Sagnes, A. Lemaître, A. V. Kavokin, G. Malpuech, J. Bloch, Propagation and amplification dynamics of 1D polariton condensates. *Phys. Rev. Lett.* **109**, 216404 (2012).

52. Y. Li, G. I. Martone, L. P. Pitaevskii, S. Stringari, Superstripes and the excitation spectrum of a spin-orbit-coupled Bose-Einstein condensate. *Phys. Rev. Lett.* **110**, 235302 (2013).

53. R. Kanamoto, L. D. Carr, M. Ueda, Metastable quantum phase transitions in a periodic one-dimensional Bose gas: Mean-field and Bogoliubov analyses. *Phys. Rev. A* **79**, 063616 (2009).

54. H. M. Price, M. Wimmer, M. Monika, U. Peschel, I. Carusotto, Theory of hydrodynamic phenomena in optical mesh lattices. *Phys. Rev. A* **108**, 063517 (2023).

55. C. N. Weiler, T. W. Neely, D. R. Scherer, A. S. Bradley, M. J. Davis, B. P. Anderson, Spontaneous vortices in the formation of Bose–Einstein condensates. *Nature* **455**, 948–951 (2008).

56. D. D. Solnyshkov, A. V. Nalitov, G. Malpuech, Kibble-Zurek mechanism in topologically nontrivial zigzag chains of polariton micropillars. *Phys. Rev. Lett.* **116**, 046402 (2016).

57. D. Solnyshkov, R. Mirek, D. Urbonas, E. Kobiyama, P. Tassan, I. Georgakilas, R. F. Mahrt, M. Forster, U. Scherf, M. Muszynski, W. Piecek, P. Kapuscinski, J. Szczytko, T. Stoferle, G. Malpuech, Kibble-Zurek mechanism in a polariton supersolid. arXiv:2512.17362 [cond-mat.mes-hall] (2025).

58. D. W. Berreman, Optics in stratified and anisotropic media: 4 × 4-matrix formulation. *J. Opt. Soc. Am.* **62**, 502–510 (1972).

59. A. V. Kavokin, M. A. Kaliteevski, Excitionic light reflection and absorption in semiconductor microcavities at oblique incidence. *Solid State Commun.* **95**, 859–862 (1995).

60. V. Savona, L. C. Andreani, P. Schwendimann, A. Quattropani, Quantum well excitons in semiconductor microcavities: Unified treatment of weak and strong coupling regimes. *Solid State Commun.* **93**, 733–739 (1995).

61. M. Kuwata-Gonokami, N. Saito, Y. Ino, M. Kauranen, K. Jefimovs, T. Vallius, J. Turunen, Y. Svirko, Giant optical activity in quasi-two-dimensional planar nanostructures. *Phys. Rev. Lett.* **95**, 227401 (2005).

62. E. Sedov, M. M. Glazov, P. G. Lagoudakis, A. V. Kavokin, Light-matter coupling and spin-orbit interaction of polariton modes in liquid crystal optical microcavities. *Phys. Rev. Res.* **6**, 023220 (2024).

63. I. A. Shelykh, Y. G. Rubo, G. Malpuech, D. D. Solnyshkov, A. Kavokin, Polarization and propagation of polariton condensates. *Phys. Rev. Lett.* **97**, 066402 (2006).

64. D. D. Solnyshkov, Hybrid Boltzmann–Gross-Pitaevskii theory of Bose-Einstein condensation and superfluidity in open driven-dissipative systems. *Phys. Rev. A* **89**, 033626 (2014).

65. L. P. Pitaevskii, Phenomenological theory of superfluidity near the λ point. *Sov. Phys. JETP* **8**, 282 (1959).

66. M. Vladimirova, S. Cronenberger, D. Scalbert, K. V. Kavokin, A. Miard, A. Lemaître, J. Bloch, D. Solnyshkov, G. Malpuech, A. V. Kavokin, Polariton-polariton interaction constants in microcavities. *Phys. Rev. B* **82**, 075301 (2010).

67. A. C. Berry, The accuracy of the Gaussian approximation to the sum of independent variates. *Trans. Am. Math. Soc.* **49**, 122–136 (1941).

68. C.-G. Esseen, A moment inequality with an application to the central limit theorem. *Scand. Actuar. J.* **1956**, 160–170 (1956).

69. H. Souissi, M. Gromovyi, I. Septembre, V. Develay, C. Brimont, L. Doyennette, E. Cambril, S. Bouchoule, B. Alloing, E. Frayssinet, J. Zúñiga-Pérez, T. Ackemann, G. Malpuech, D. D. Solnyshkov, T. Guillet, Mode-locked waveguide polariton laser. *Optica* **11**, 962–970 (2024).
